# Integrative Phytochemical, Morphological and Molecular Analyses Reveal Intraspecific Diversification in Wild Iranian Populations of *Heracleum persicum* Desf. ex Fischer

**DOI:** 10.3390/plants15203200

**Published:** 2026-10-19

**Authors:** Sima Sadat Rafiei, Kazem Mahdigholi, Mohsen Falahati-Anbaran

**Affiliations:** 1 Department of Plant Biology, School of Biology, College of Science, University of Tehran, Tehran 14179-35840, Iran; 2 Department of Natural History, NTNU University Museum, Norwegian University of Science and Technology, 7491 Trondheim, Norway

**Keywords:** *Heracleum persicum*, leaf chemical profiles, fruit morphometrics, GC-MS analysis, molecular markers, ecological gradients

## Abstract

*Heracleum persicum* Desf. ex Fischer is an aromatic and medicinal plant with substantial natural variation, yet its population-level differentiation remains poorly resolved. We applied an integrative framework combining leaf chemical profiling (GC–MS), fruit morphometrics, multilocus molecular markers (ITS, *psb*A–*trn*H, *rpl*32–*trn*L), and ecological gradients to investigate intraspecific differentiation among 13 wild populations. We identified 90 chemical compounds belonging to 15 chemical classes, with substantial variation in fatty acids and aliphatic hydrocarbons. Multivariate analyses revealed three distinct chemical groups, including populations enriched in unsaturated fatty acids, a group associated with saturated fatty acids and alkanes, and the highly differentiated Zeri population characterized by elevated anethole. Fruit morphological traits and molecular relationships showed correspondence with chemical differentiation, particularly in the Margoon–Komehr and Alashtar–Kamandan–Naran groups. Canonical correspondence analysis indicated associations between chemical composition and altitude, temperature, and precipitation. Overall, the correspondence among phytochemical, morphological, molecular, and ecological datasets demonstrates substantial geographically structured intraspecific differentiation in *H. persicum*. These findings highlight the value of integrating independent character systems for recognizing and characterizing population lineages and provide a biosystematic framework for understanding natural variation within *H. persicum*.

## 1. Introduction

The genus *Heracleum* (Apiaceae) comprises more than 125 species distributed mainly across the Northern Hemisphere, with Asia representing its major diversity center [1,2,3,4]. In Iran, nine species are recognized [5,6,7,8], among which *Heracleum persicum* Desf. ex Fischer is an economically important aromatic and medicinal plant traditionally used for culinary and therapeutic purposes including gastrointestinal and neurological applications [9,10,11,12,13].

Taxonomic complexity within *Heracleum* complicates the use of morphology alone for population or species delimitation. Morphological overlap, incongruence between nuclear and plastid phylogenies, and evidence of hybridization and incomplete lineage sorting underscore the need for integrative approaches [1,14,15,16,17,18,19]. Fruit and seed micromorphology provide valuable taxonomic characters [2,20,21,22], but their resolution is limited when used independently. Molecular studies have revealed geographically structured genetic lineages in Iranian *H. persicum* populations [23], while other analyses report weak differentiation among closely related species [24]. For *H. persicum*, previous studies have documented substantial variation in essential oil composition of fruits among Iranian populations, with climatic and edaphic factors contributing to differences in the abundance of key chemical compounds [25,26]. Population-specific chemical profiles, including distinct anethole chemotypes, further indicate pronounced intraspecific chemical differentiation across the species’ geographic range [25]. Taken together, these findings highlight substantial intraspecific variation across chemical, morphological, molecular, and ecological traits. Although essential oils from fruits of *H. persicum* have been widely studied [26,27,28,29,30,31,32,33], population-level variation in leaf chemical composition and their relationship with morphology, genetic, and ecological gradients has not been evaluated simultaneously.

Integrative biosystematics studies combining phytochemical, morphological, molecular, and ecological evidence can identify patterns of population differentiation and intraspecific diversification associated with distinct chemotypes, morphological traits, and genetic backgrounds. Such an integrative approach can provide a more comprehensive understanding of diversification patterns and drivers of intraspecific differentiation in *H. persicum*. Here, we present an integrative assessment of leaf chemical compounds, fruit morphological variation, molecular phylogeny, and ecological differentiation across 13 natural populations of *H. persicum*. Specifically, we aimed to (i) characterize population-level variation in leaf chemical composition, (ii) evaluate fruit morphological and morphometric diversity, (iii) reconstruct genetic relationships using nuclear and plastid markers, and (iv) assess associations between phytochemical variation and climatic variables. By integrating these independent lines of evidence, we aim to characterize patterns of geographic and ecological population differentiation and to evaluate the extent to which morphological, molecular, and phytochemical variation correspond across the natural range of *H. persicum*.

## 2. Results

### 2.1. GC-MS Profiling of Derivatized Leaf Samples

**Figure 1:**
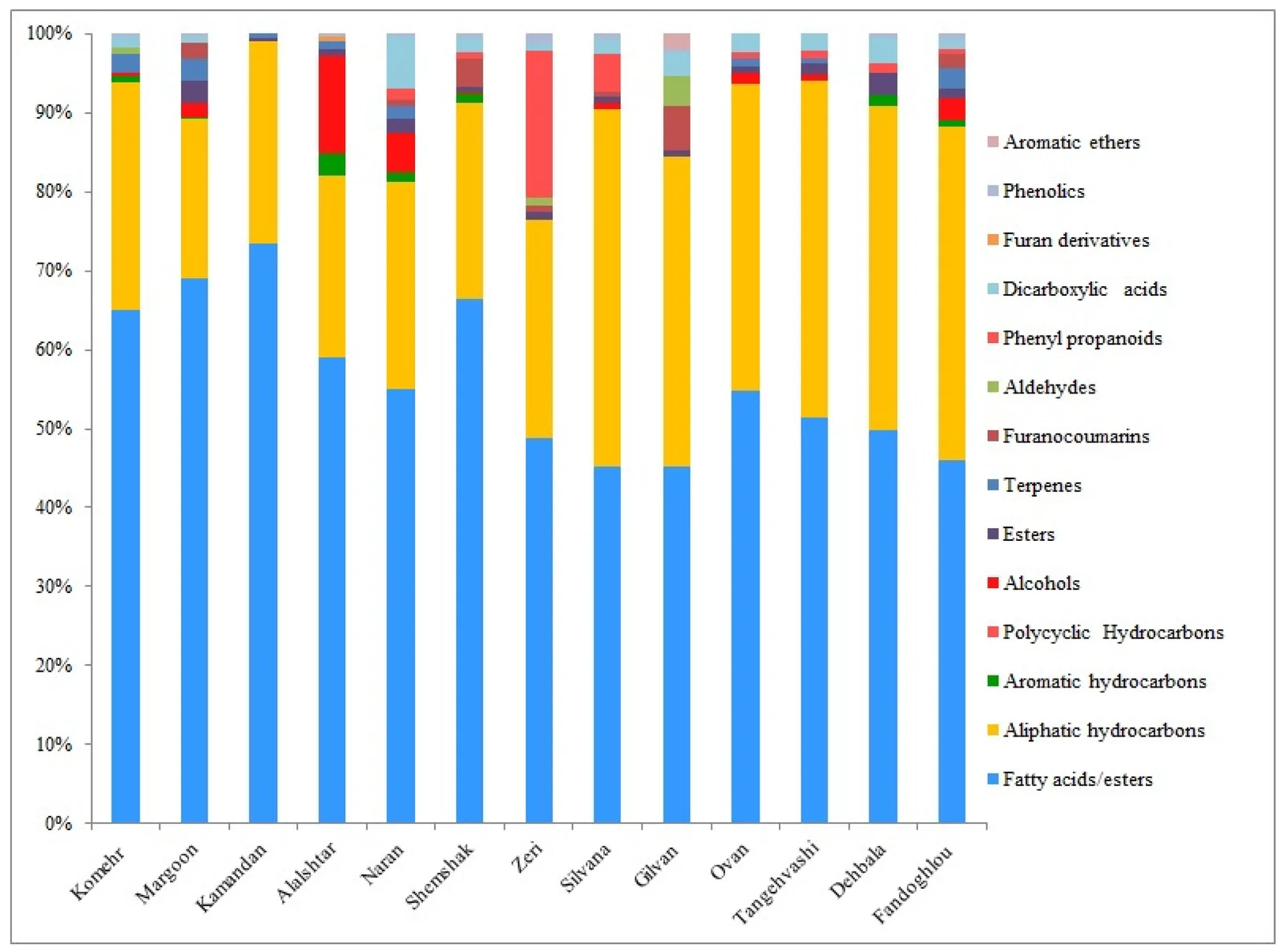
Relative distribution of identified chemical classes in 13 populations of *H. persicum*.

GC–MS analysis identified 90 chemical compounds across the 13 *H. persicum* populations, representing 15 chemical classes and accounting for 90.44–98.18% of the total content of chemical compounds (Table 1; Figure 1). Fatty acids dominated all profiles (44.01–70.30%), with α-linolenic acid, linoleic acid, and roughanic acid representing the major polyunsaturated fatty acids. Populations such as Kamandan, Komehr, Margoon, Alashtar, Shemshak, and Zeri exhibited more than 20% α-linolenic acid, whereas linoleic acid exceeded 10% in several populations. Palmitic acid was consistently abundant across populations (13.8–23.55%), whereas stearic and myristic acids occurred at lower but variable proportions. Aliphatic hydrocarbons constituted the second major chemical class (19.82–44.34%), with decane, dodecane, and tetradecane being among the predominant compounds. Fumaric acid was the only dicarboxylic acid detected and reached its highest relative abundance in Naran (6.06%) (Table 1). Overall, the chemical profiles showed pronounced quantitative variation among the 13 populations, with differences in the relative abundance of fatty acids and aliphatic hydrocarbons.

### 2.2. Among-Population Variation in Chemical Profiles

**Figure 2:**
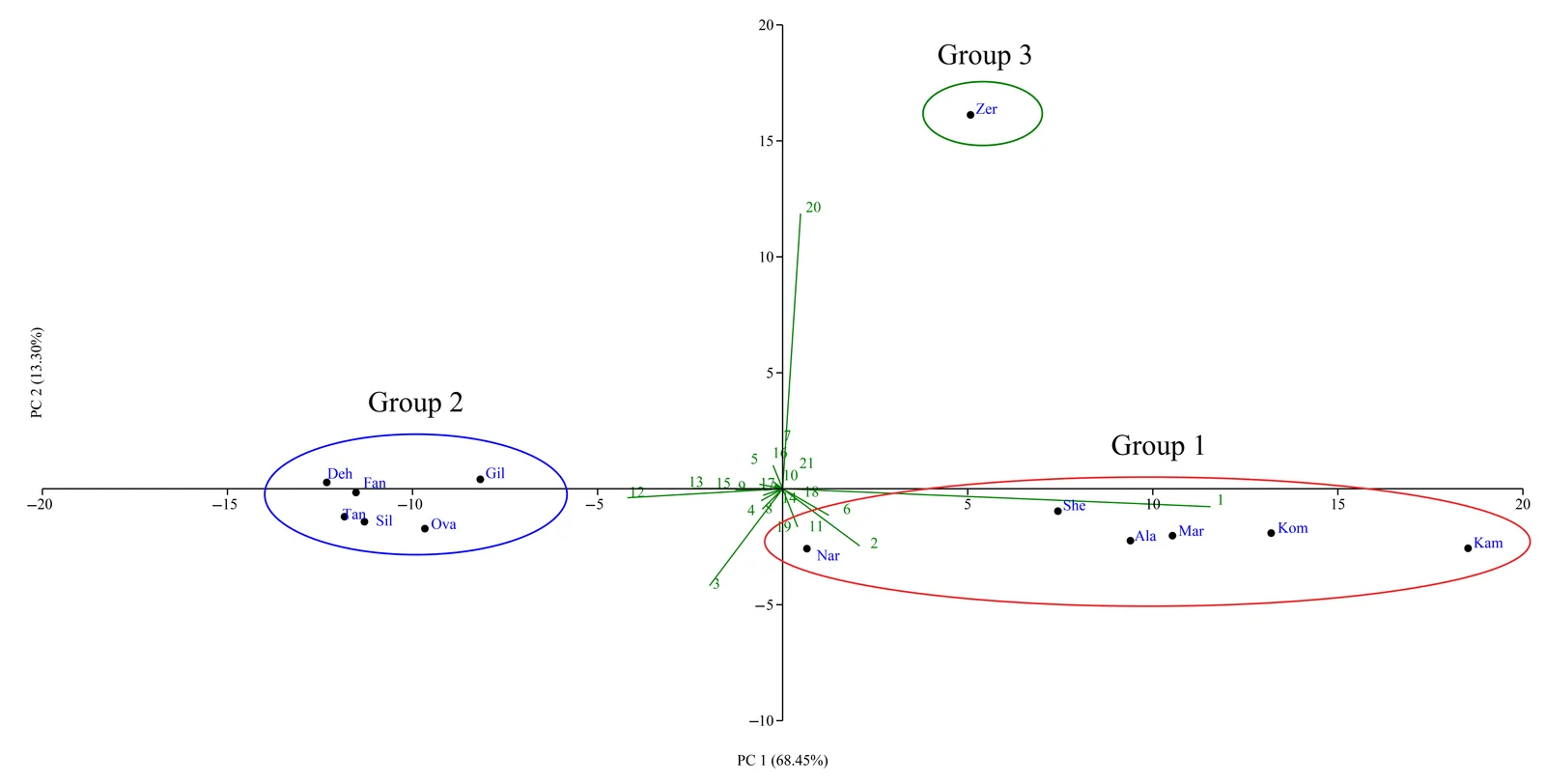
Principal component analysis (PCA) based on 21 major chemical compounds. Compounds abbreviations: 1: α-Linolenic acid; 2: Linoleic acid; 3: Palmitic acid; 4: Stearic acid; 5: Myristic acid; 6: Roughanic acid; 7: Palmitoleic acid; 8: Behenic acid; 9: Lignoceric acid; 10: Nonane, 3-methyl-; 11: 9,12,15-Octadecatrien-1-ol, (Z,Z,Z)-; 12: Decan; 13: Dodecane; 14: Tridecane; 15: Tetradecane; 16: Hexadecane; 17: Octadecane; 18: Undecane; 19: Fumaric acid; 20: Anethole; 21: Neophytadiene. See Table 3 for population abbreviations.

**Figure 3:**
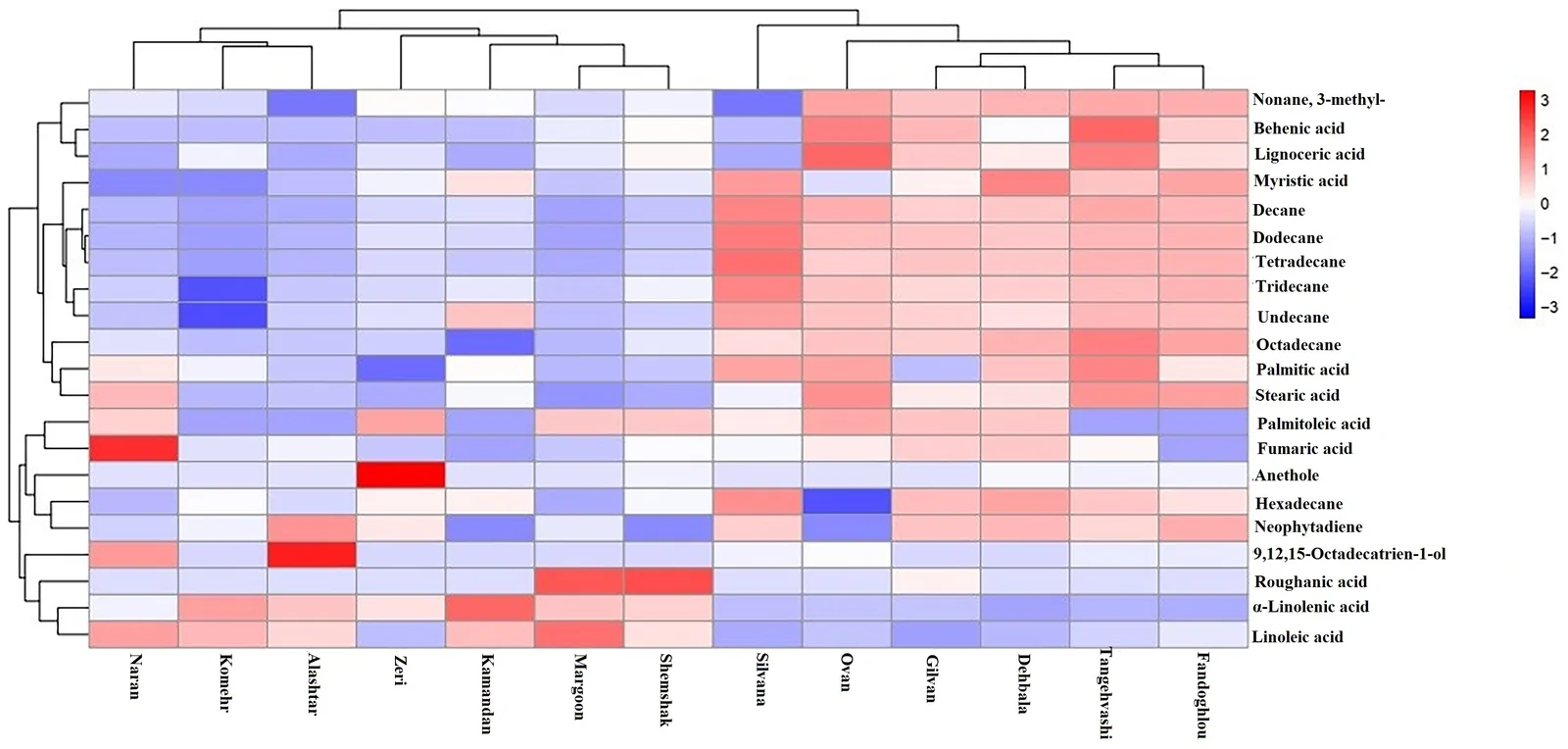
Heat-map diagram of two-way hierarchical cluster analysis (HCA) of thirteen *H. persicum* populations based on 21 major chemical compounds quantified by GC–MS. Red color with a great positive share and blue color with a great negative share affects cluster formation.

Principal component analysis revealed clear differentiation among the studied populations, with PC1 and PC2 together explaining 81.75% of the total variation (Figure 2). A similar result was observed by including all 90 identified compounds in the PCA (Figure S1). The ordination analysis separated the populations into three major chemical groups. Kamandan, Komehr, Margoon, Alashtar, Shemshak, and Naran clustered together and were characterized by elevated levels of unsaturated fatty acids, particularly α-linolenic and linoleic acids. In contrast, Fandoghlou, Silvana, Ovan, Tangehvashi, Dehbala, and Gilvan formed a second group associated with higher concentrations of saturated fatty acids and aliphatic hydrocarbons. Zeri occupied a clearly separated position in the PCA space, primarily associated with its exceptionally high anethole content. Hierarchical cluster analysis further supported the chemical differentiation observed in the PCA, separating the populations into two major clusters, with Zeri positioned separately from the other populations (Figure 3). Together, the PCA and cluster analyses revealed pronounced population-level structuring of the chemical profiles and identified Zeri as the most chemically distinctive population.

### 2.3. Correlation Among Chemical Compounds

Correlation analysis revealed structured associations among the major chemical compounds (Figure S2). Polyunsaturated fatty acids, particularly α-linolenic and linoleic acids, showed a positive correlation (*r* = 0.67). These compounds were negatively correlated with most alkanes. Saturated fatty acids formed a second correlated group, with positive associations among palmitic, stearic, behenic, and lignoceric acids (*r* = 0.55–0.75). Aliphatic hydrocarbons, including tetradecane, decane, and dodecane, exhibited very strong positive correlations, with correlation coefficients ranging from 0.99 to 1.00. The correlation analysis therefore identified distinct groups of co-varying compounds corresponding broadly to polyunsaturated fatty acids, saturated fatty acids, and aliphatic hydrocarbons.

### 2.4. Associations Between Chemical Profiles and Ecological Variables

**Figure 4:**
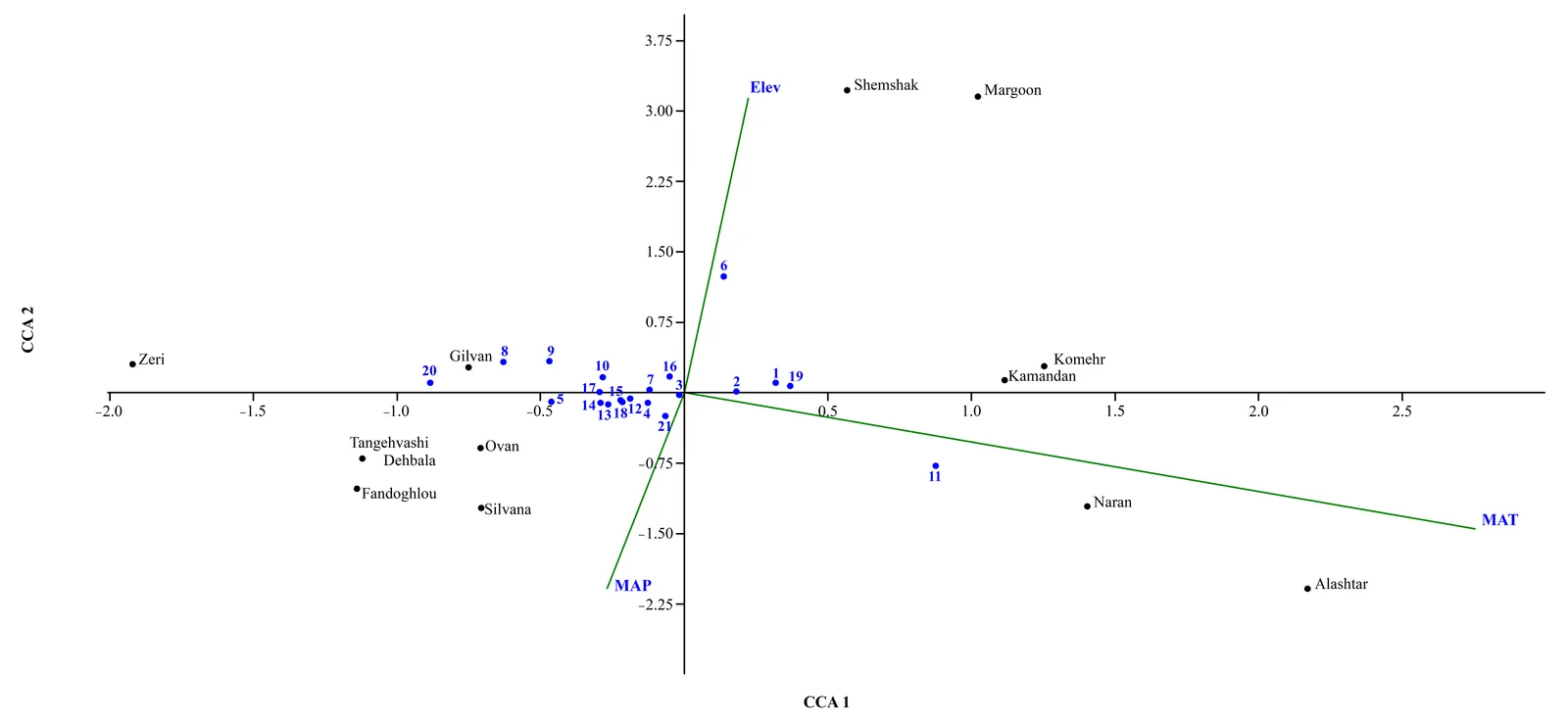
Canonical correspondence analysis biplot of *H. persicum* populations, linking percentages of the major components, collected site and their climatic characteristics. See Figure 2 for compound abbreviations. Abbreviations for environmental variables: Elev: Elevation; MAT: Mean Annual Temperature; MAP: Mean Annual Precipitation.

Canonical Correspondence Analysis (CCA) revealed associations between chemical composition and the environmental variables altitude, mean annual precipitation (MAP), and mean annual temperature (MAT) (Figure 4, Table S1). The overall CCA model explained 29.08% of the total variance (constrained inertia = 0.1266; total inertia = 0.4355). The first two CCA axes accounted for 26.15% of the total variance (CCA1 = 17.13%, CCA2 = 9.02%) (Table S1). The permutation test (999 unrestricted permutations) indicated that the overall CCA model was not statistically significant (F = 1.23, *p* = 0.253). Similarly, none of the individual environmental factors showed a significant effect: elevation (F = 1.09, *p* = 0.401), MAT (F = 1.31, *p* = 0.244), and MAP (F = 1.29, *p* = 0.305). Correlation analysis was also performed to confirm the patterns revealed by CCA, and the results were broadly consistent with the ordination output (Table S2). Margoon and Shemshak were associated with higher values of roughanic acid and occurred at relatively higher elevations. Komehr and Kamandan were associated with α-linolenic acid, linoleic acid, and fumaric acid, However, none of the chemical compounds showed a statistically significant positive correlation with climatic variables (MAT and MAP). Zeri occupied a distinct position in the CCA ordination and was primarily associated with its high anethole content. Tangehvashi, Dehbala, Ovan, Fandoghlou, and Silvana were associated with aliphatic hydrocarbons and saturated fatty acids and showed positive relationships with precipitation. In addition, 9,12,15-octadecatrien-1-ol showed a positive relationship with temperature and was associated with Naran and Alashtar. Overall, although the CCA model was not statistically significant, the ordination revealed exploratory patterns suggesting that some chemical compounds may be associated with geographic and climatic gradients, with different populations occupying distinct positions along the major environmental axes. However, these associations should be considered preliminary and require confirmation with larger sample sizes and independent datasets.

### 2.5. Morphological Differentiation Based on Fruit Characters

**Figure 5:**
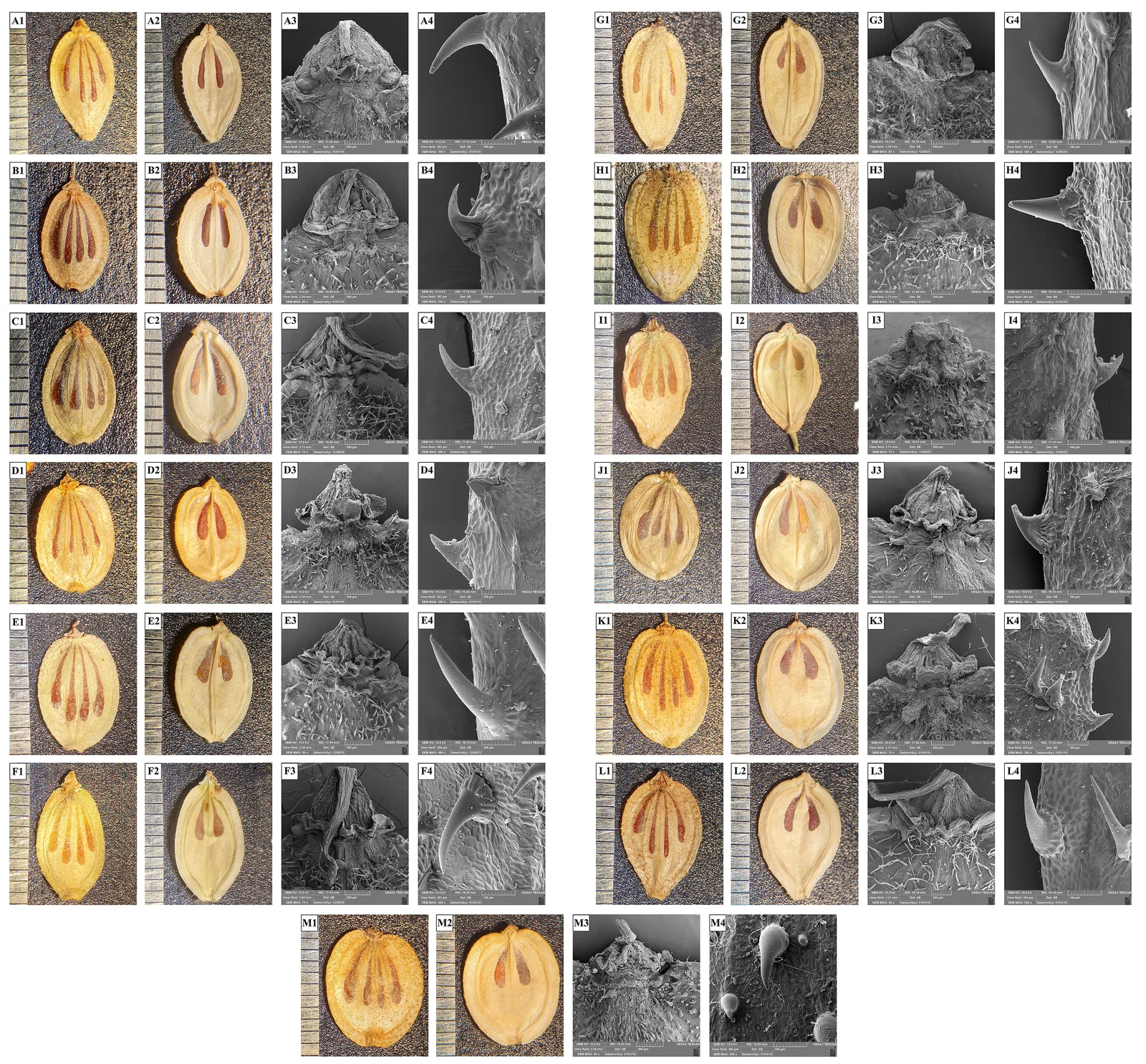
LM and SEM photographs of *H. persicum* populations fruits (mericarps) (dorsal view, commissural view, stylopodium shape, marginal trichome shape). (**A1**–**A4**: Alashtar), (**B1**–**B4**: Kamandan), (**C1**–**C4**: Margoon), (**D1**–**D4**: Komehr), (**E1**–**E4**: Naran), (**F1**–**F4**: Zeri), (**G1**–**G4**: Silvana), (**H1**–**H4**: Gilvan), (**I1**–**I4**: Shemshak), (**J1**–**J4**: Tangehvashi), (**K1**–**K4**: Ovan), (**L1**–**L4**: Dehbala), (**M1**–**M4**: Fndoghlou).

Fruit morphology exhibited substantial inter-population variation in mericarp dimensions, stylopodium shape, trichome density, and vitta structure (Figure 5; Table 2).

**Figure 6:**
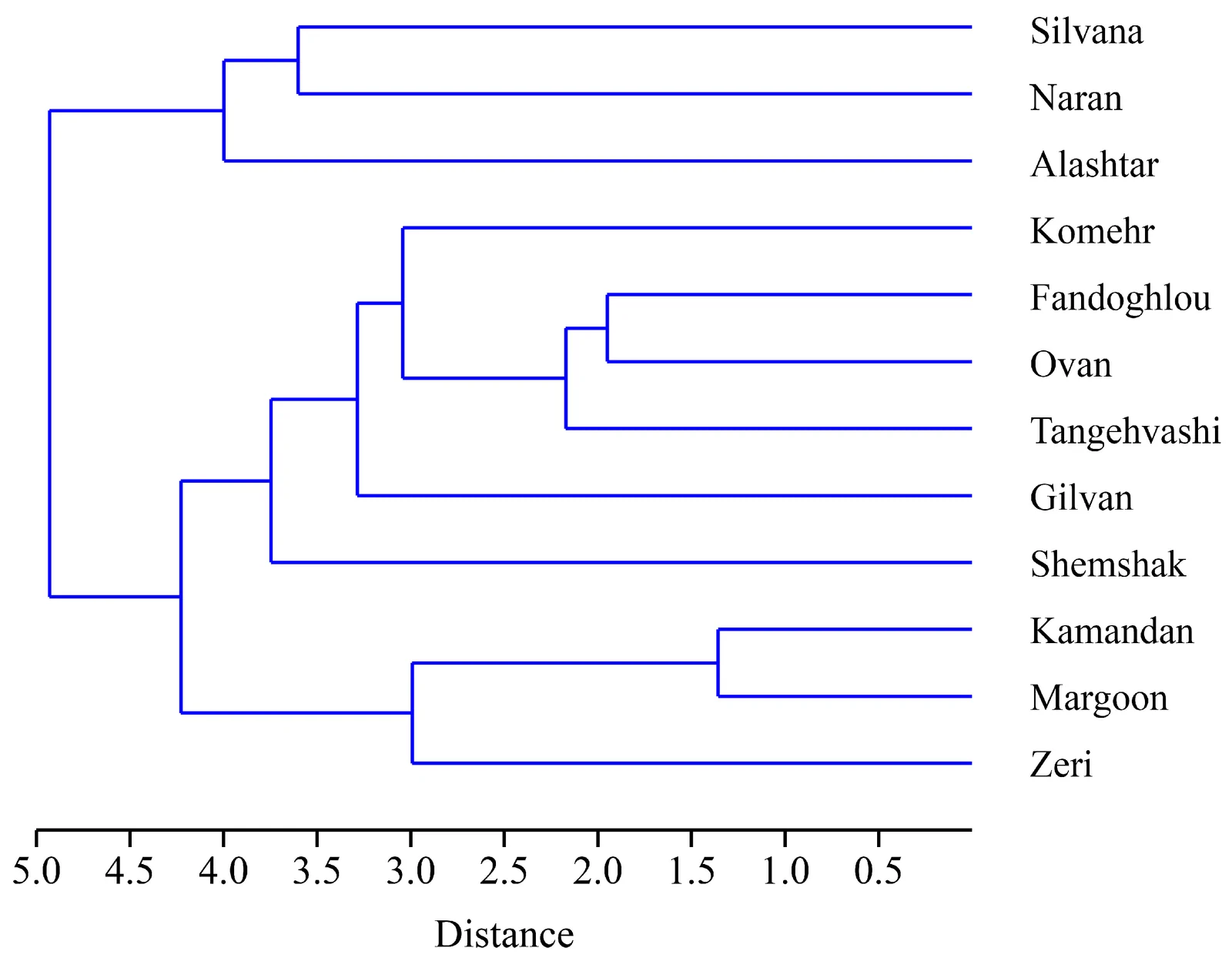
Cluster dendrogram of 13 wild populations of *H. persicum* based on fruit morphological traits.

Although all populations shared the characteristic fruit organization of *Heracleum* described in Flora Iranica [7] and subsequent taxonomic treatments [45, 22], quantitative differences were observed among populations. Stylopodium angle ranged from 71.7° in Zeri to 114.5° in Tangehvashi (Figure 5: F3,J3). Vitta morphology also varied among populations, with Margoon exhibiting swollen dorsal vittae and Komehr showing slender clavate vittae. Zeri was characterized by longer mericarps and a narrowly conical stylopodium. Principal component analysis explained 62.75% of the total morphological variation, with fruit width (FW), vitta dimensions, and stylopodium angle (SA) contributing strongly to population separation (Figure S3). The PCA distinguished several population groups, including Silvana and Naran, which were separated by higher fruit length-to-width ratios and more developed inner dorsal vittae, and Kamandan and Zeri, which were characterized by longer dorsal outer vittae and wider stylopodia. Fandoghlou and Tangehvashi grouped together, whereas Komehr and Shemshak occupied intermediate positions. Cluster analysis supported the PCA results and identified two major morphological groups with several subclusters (Figure 6). Silvana, Naran, and Alashtar formed one group, whereas Komehr, Fandoghlou, Ovan, Tangehvashi, and Gilvan formed another. Kamandan, Margoon, and Zeri clustered together based on their larger dorsal vittae and more developed stylopodia.

These results demonstrate substantial population-level differentiation in fruit morphology, particularly in fruit dimensions, vitta characters, and stylopodium morphology.

### 2.6. Phylogenetic Relationships Among Populations

**Figure 7:**
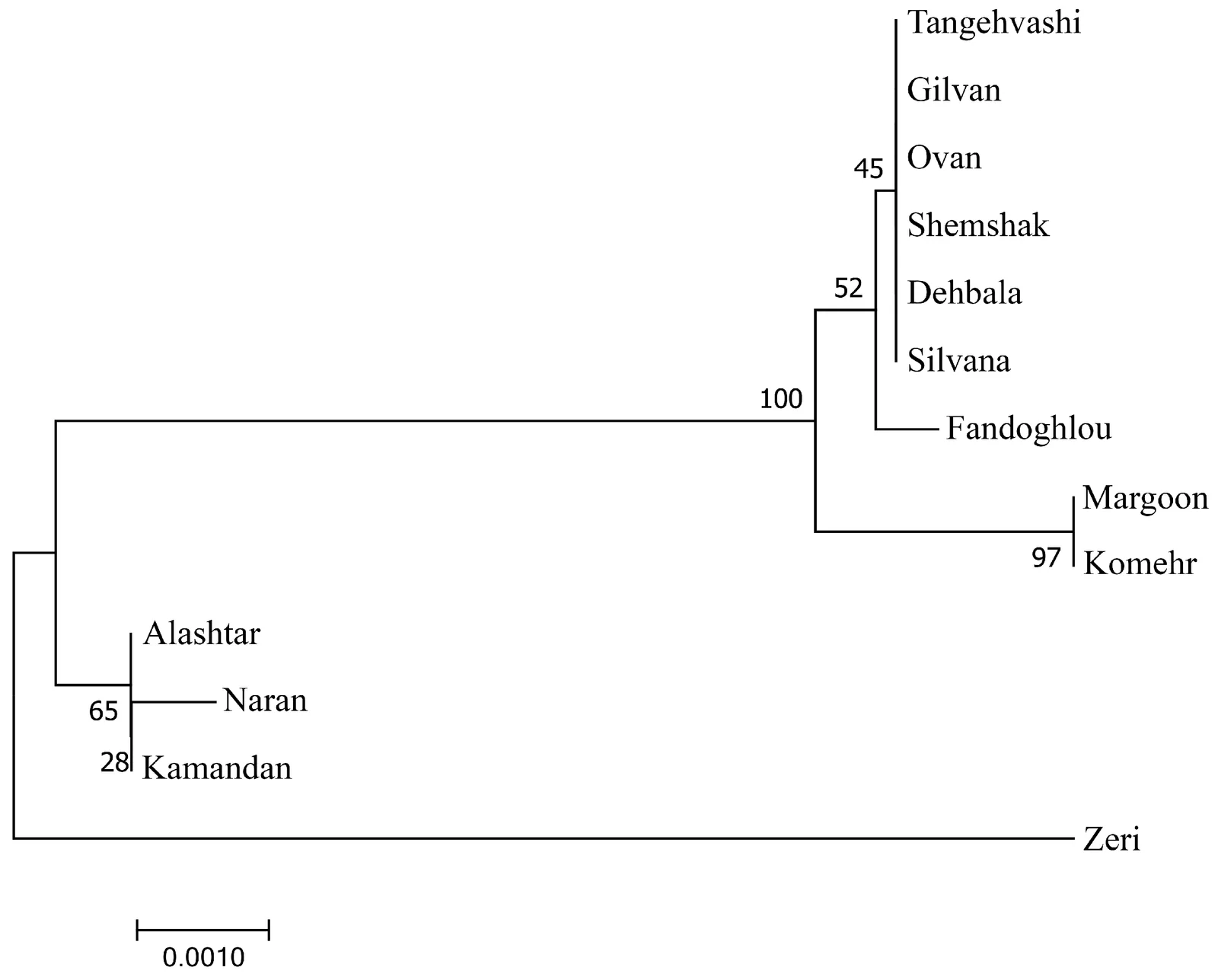
Neighbor-Joining (NJ) phylogenetic tree inferred from the combined ITS, *psb*A-*trn*H, and *rpl*32-*trn*L sequence dataset of 13 wild populations of *H. persicum*. Number on the branches represent bootstrap support obtained by 1000 bootstrap replicates.

Neighbor-Joining analysis of the combined ITS, *psb*A–*trn*H, and *rpl*32–*trn*L nucleotide sequence dataset revealed two major genetic groups among the 13 populations, with Zeri occupying a distinct position (Figure 7). Margoon and Komehr formed a strongly supported sister group (BS = 97), and this pair was sister to a larger assemblage including Fandoghlou, Silvana, Dehbala, Shemshak, Ovan, Gilvan, and Tangehvashi (BS = 100). A second group comprised Alashtar, Kamandan, and Naran (BS = 65). Zeri was positioned separately from the two major groups. Comparison with the phylogeographic framework of Falahati-Anbaran et al. showed partial correspondence [23]. Dehbala, Shemshak, Ovan, and Tangehvashi were clustered within the same major lineage, while Margoon, Komehr, Alashtar, and Kamandan showed correspondence with previously reported genetic patterns. Naran was positioned close to Alashtar and Kamandan. In contrast, Zeri showed a different placement from that reported previously, where it was assigned to the western lineage. The distinct phylogenetic position of Zeri, reflected in its long branch length, coincided with its marked differentiation in both fruit morphology and chemical composition.

## 3. Discussion

### 3.1. Chemical Differentiation Among Natural Populations

The present study revealed pronounced chemical differentiation among natural populations of *H. persicum*, indicating that leaf chemical composition represents a variable character system within the species. The clear separation of populations in multivariate analyses, together with the consistency between PCA and HCA, indicates that this variation is structured rather than randomly distributed. The observed chemical differentiation was mainly associated with variation in fattyacid composition and the relative contribution of aliphatic and aromatic compounds, with distinct chemical profiles characterizing different groups of populations.

The predominance of fatty acids in derivatized leaf samples is consistent with the important role of these compounds in plant metabolism. In particular, polyunsaturated fatty acids such as α-linolenic and linoleic acids are involved in maintaining membrane stability under environmental stress and serve as precursors of signaling molecules involved in plant growth and stress responses [34]. The marked differences in their relative abundance among populations therefore provide evidence of substantial biochemical differentiation within *H. persicum*. Similar compounds have been reported in leaves of other species from Apiaceae, although generally at lower levels [35], and in *Heracleum sosnowskyi* [36]. The present findings therefore add to the limited information available on the leaf chemical composition of *Heracleum*.

The substantial variation in aliphatic hydrocarbons represents another important component of the observed chemical differentiation. Decane, dodecane, and tetradecane contributed considerably to the profiles of several populations and showed different levels among them. Such compounds potentially related to plant surface lipids and cuticular waxes through their biosynthetic origin [37,38]. Their variable representation among populations, together with differences in fattyacid composition, contributed to the separation of populations observed in the multivariate analyses. The chemical differentiation detected in the present study is broadly consistent with previous evidence of population-specific chemical variation in *H. persicum*. Falahati-Anbaran et al. demonstrated considerable variation in essential oil composition of fruit among Iranian populations and reported geographically structured differences in major chemical compounds, including variation in the hexyl butyrate/octyl acetate ratio and population-specific occurrence of high anethole levels with potential taxonomic relevance [25]. Overall, the results of the PCA and HCA analyses are consistent with each other in showing the chemical heterogeneity of the populations. Both diagrams show that the observed differences are not only due to random changes in one compound, but rather a set of hydrocarbons, fatty acids, and some specific metabolites play a role in the separation of the populations. The clearest agreement between the two analyses concerns the Zeri population. In the heatmap, this population shows a very high level of anethole, and in the PCA biplot, Zeri is positioned along the anethole vector and at a large distance from the other populations. Therefore, it can be confidently concluded that the high level of anethole is the most important factor in the chemical differentiation of Zeri. The correspondence between the present chemical patterns and previously reported genetic variation is particularly relevant from a biosystematic perspective. Several populations showing similar chemical profiles in the present study were also associated with the western genetic clade identified by Falahati-Anbaran et al. [23] Moreover, the relationship between the genetic patterns reported by Falahati-Anbaran et al. [23] , the fruit-based chemical patterns reported by Falahati-Anbaran et al. [25], and the leaf chemical profiles observed here provides evidence that different characters can show correspondence in the differentiation of natural populations.

### 3.2. Association Between Phytochemical Variation and Ecological Factors

The observed relationships between chemical composition and ecological variables suggest that phytochemical variation among natural populations of *H. persicum* may be structured along environmental gradients. The exploratory patterns observed in CCA explains that ecological variables including altitude, mean annual temperature (MAT), and mean annual precipitation (MAP) are associated with differences in the chemical profiles of the populations (Figure 4). However, the overall CCA model and individual environmental factors were not statistically significant, and these patterns should therefore be interpreted with caution. Despite this limitation, the association of Margoon and Shemshak with roughanic acid may suggest that elevation could contribute to differences in the accumulation of this compound. Higher elevations are characterized by lower temperatures, and plants adapt to such conditions by increasing the degree of unsaturated fatty acid, which helps retain the fluidity of cell membranes in the physiological range at low temperatures [39]. The associations observed for Komehr and Kamandan were characterized by α-linolenic acid, linoleic acid, and fumaric acid, which showed negative relationships with temperature. The association of these unsaturated fatty acids with populations occurring under relatively cooler conditions is consistent with their established role in maintaining membrane fluidity under low-temperature conditions [40]. The similar chemical profiles of Komehr and Kamandan also correspond with their previously reported genetic similarity [23], suggesting that the observed phytochemical patterns may reflect the combined contribution of ecological and genetic differentiation among populations. Zeri again occupied a distinct position in the CCA, mainly associated with its high anethole content. This distinctive chemical profile, together with its separation in the ecological ordination, further emphasizes the pronounced phytochemical differentiation of this population. In contrast, Tangehvashi, Dehbala, Ovan, Fandoghlou, and Silvana were associated with alkanes and saturated fatty acids. Although these populations showed a tendency toward higher precipitation, the relationship remains unclear. The alkanes detected here (C_10_–C_18_) are shorter than typical wax VLCFAs (C_20_–C_34_), suggesting they may be by-products of incomplete elongation rather than functional cuticular components. No direct evidence links medium- to long-chain alkanes to precipitation, and precipitation–alkane relationships vary across studies. These patterns are consistent with a potential influence of environmental gradients on alkane and saturated fatty acid accumulation, although further studies are needed to confirm this relationship [38,41].

The positive relationship between 9,12,15-octadecatrien-1-ol and temperature, particularly its association with Naran and Alashtar, further suggests that individual lipid-related compounds may vary along climatic gradients. Taken together, although the CCA model was not statistically significant, the results suggest that the chemical composition of natural *H. persicum* populations may be associated with ecological differentiation, particularly along gradients of altitude, temperature, and precipitation. These relationships provide exploratory evidence for characterizing population-level phytochemical differentiation and contribute to the biosystematics interpretation of variation within *H. persicum*. However, they should be considered preliminary and require confirmation with larger sample sizes, multi-season sampling, and independent datasets.

### 3.3. Fruit Morphological Differentiation and Its Taxonomic Significance

The taxonomy of *Heracleum* has long been considered problematic because of considerable morphological overlap among species, making species delimitation difficult when based solely on morphological characters. Molecular studies have also shown that morphological groups are not always congruent with evolutionary relationships, emphasizing the importance of integrating morphological and molecular evidence in systematic studies [18,22]. Fruit morphology has traditionally been an important source of taxonomic information in *Heracleum*, contributing to species identification and the delimitation of infrageneric groups [21]. In particular, fruit and seed anatomical and micromorphological characters have been shown to be taxonomically informative and may correspond with major molecular clades, although they are insufficient for reconstructing phylogenetic relationships independently [22]. Likewise, mericarp shape, vittae configuration, stylopodium morphology, and trichome characters provide useful diagnostic information for systematic studies within *Heracleum* [20]. In the present study, substantial variation in mericarp dimensions, vitta morphology, and stylopodium characters was observed among the studied populations. Fruit width, vitta dimensions, and stylopodium angle contributed most strongly to the observed morphological separation in the PCA. These findings support the taxonomic usefulness of fruit characters for assessing morphological differentiation within *H. persicum*, in agreement with previous studies of *Heracleum* [20,22].

The morphological distinctiveness of Zeri is particularly noteworthy because this population also showed pronounced differentiation in the phytochemical and molecular datasets. This correspondence among independent character sets provides additional support for the usefulness of fruit morphology in characterizing population-level differentiation. Overall, the observed variation in mericarp, vitta, and stylopodium characters demonstrates that fruit morphology provides a useful component of an integrative biosystematics assessment of intraspecific variation in *H. persicum*.

### 3.4. Phylogenetic Differentiation and Population Structure

The combined analysis of the nuclear ITS and plastid *psb*A–*trn*H and *rpl*32–*trn*L markers revealed structured genetic variation among the studied populations of *H. persicum*. The recovered relationships showed relatively close affinities among several geographically associated populations, including the strongly supported Margoon–Komehr pair (BS = 97) and the Alashtar–Kamandan–Naran group (BS = 65). These results are broadly consistent with previous molecular evidence demonstrating geographically structured genetic variation among Iranian populations of *H. persicum* [23].

Previous phylogeographic analyses divided Iranian populations of *H. persicum* into western, central, and eastern lineages [23]. The partial correspondence between this framework and the present phylogeny suggests that geographic structure contributes to genetic differentiation within the species, although the relationships among populations are not completely resolved according to geographic regions. In particular, the close association of Margoon and Komehr in the present analysis is consistent with their affinity within the previously reported central lineage. Likewise, the association of Alashtar and Kamandan with Naran provides additional evidence for genetic differentiation among geographically structured populations.

Notably, the distinct position of the Zeri population was not confined to the molecular dataset; it was also consistently recovered in the phytochemical (GC-MS-derived profiles), morphological (morphometric traits), and ecological (altitude, MAT, MAP) datasets, suggesting a structured and congruent pattern of differentiation. The placement of Zeri also represents the most notable difference from the phylogeographic pattern reported by Falahati-Anbaran et al. [23]. Whereas Zeri was previously assigned to the western lineage [23], the present analysis placed it outside the two principal lineages. Previous studies have emphasized that morphological and molecular datasets may not always produce completely congruent relationships within *Heracleum*, reflecting the taxonomic complexity of the genus [18,22]. However, in the present study, this separation was largely observed, with the Zeri population showing consistent divergence across dataset. Therefore, the distinct position of Zeri, supported by congruent signals across molecular, phytochemical, and morphological datasets, highlights it as a notable case of population-level differentiation within *H. persicum*. The resolution obtained from the combined markers is also relevant in light of previous molecular studies of *Heracleum*. Phylogenetic investigations have demonstrated differences in the discriminatory power of nuclear and plastid markers among closely related taxa, with plastid regions often providing useful information for resolving relationships that are poorly differentiated by ITS alone [18]. Conversely, Eidi et al. reported limited discriminatory power of ITS and *rpl*32–*trn*L among closely related *Heracleum* taxa [42]. The resolution of several population groups in the present combined analysis therefore indicates that the use of multiple molecular markers can provide additional information on intraspecific structure, despite the relatively limited variation in individual markers. The molecular patterns also show correspondence with the other datasets examined in this study. The Margoon–Komehr and Alashtar–Kamandan–Naran groups identified molecularly were also differentiated in their phytochemical profiles, while Zeri showed the most pronounced differentiation across the molecular, morphological, and chemical datasets. Such correspondence is consistent with the value of integrating independent character systems in taxonomically complex groups, where no single dataset may fully resolve population relationships [18,22].

Overall, the molecular data confirm substantial intraspecific genetic structure among natural populations of *H. persicum* and indicate that the evolutionary relationships among these populations are more complex than a simple geographic subdivision. The observed correspondence between molecular differentiation and variation in fruit morphological traits and chemical compounds, particularly in the Margoon–Komehr, Alashtar–Kamandan–Naran, and Zeri populations, suggests that some of these populations may represent distinct intraspecific lineages. These findings highlight the importance of an integrative biosystematics approach for revealing and interpreting patterns of intraspecific differentiation and diversification in *H. persicum*.

## 4. Materials and Methods

### 4.1. Plant Material

**Figure 8:**
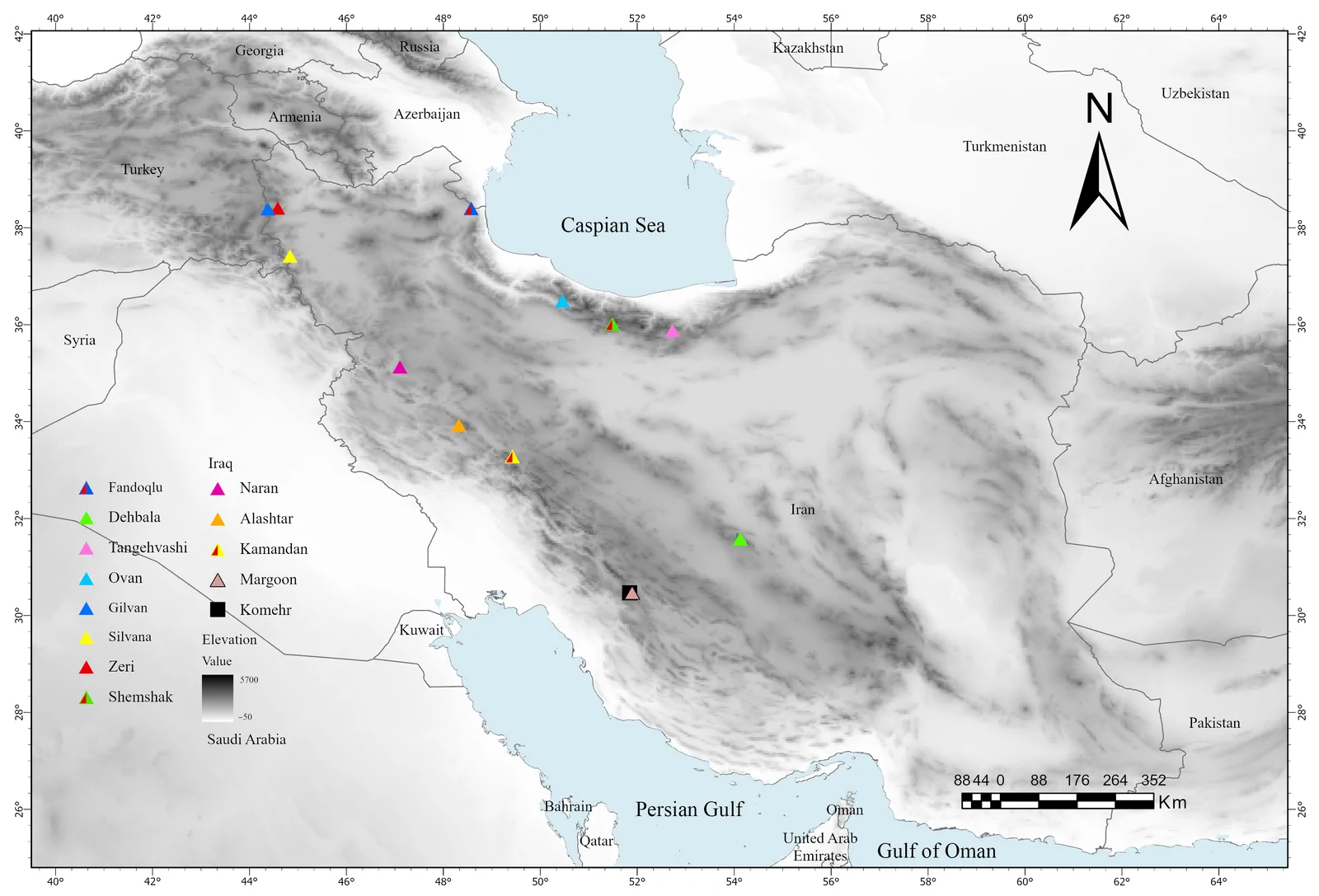
Geographic distribution of natural populations of *H. persicum* analyzed in this study.

Wild populations of *Heracleum persicum* were collected from 13 different geographical locations during June-July 2024 in Iran. The plant specimens were identified according to Flora Iranica, and voucher specimens were deposited in the Central Herbarium of the University of Tehran. For each collection site, geographic coordinates—including latitude, longitude, and elevation—were recorded using a Global Positioning System (GPS). In addition, three ecological factors were obtained for each site: elevation (Elev, m), mean annual temperature (MAT, °C), and mean annual precipitation (MAP, mm) (Table 3, Figure 8). MAT and MAP were extracted from the WorldClim global climate database (version 2.1) at a 30 arc-second resolution [43].

### 4.2. Preparation of Derivatized Leaf Samples for GC-MS Analysis

To characterize the GC-MS-derived chemical profile of leaves, the leaf samples of *H. persicum* were ground to obtain a fine powder. For each population, leaves collected from five individual plants were pooled to obtain a population-level sample. Subsequently, 0.1 g of the pooled leaf material was transferred into test tubes containing 1 mL of the methylation mixture (methanol/acetyl chloride, 20:1 *v*/*v*) and 0.5 mL hexane and incubated at 100 °C for 10 min. This procedure results in acid-catalyzed transesterification, converting fatty acids and related compounds into their corresponding methyl esters. A single methanol/hexane phase was formed. After cooling to room temperature, 1 mL distilled water was added. Two phases separated rapidly, and the upper hexanoic layer was extracted and placed into the chromatograph vial for injection [44]. A single extraction was performed for each pooled population sample, and the resulting extract was analyzed by one GC–MS injection. Thus, each population was represented by one population-level GC–MS chemical profile.

### 4.3. Gas Chromatography/Mass Spectroscopy (GC-MS) Analysis

The chemical compounds detected in the derivatized leaf samples were identified based on their molecular formula, retention time (RT) and National Institute of Standards and Technology (NIST) library similarity index. GC-MS analyses were carried out using an Agilent 7890A GC system (Agilent Technologies, Santa Clara, CA, USA) coupled with an Agilent 5975C VL MSD with Triple-Axis Detector (Agilent Technologies, Santa Clara, CA, USA) and equipped with an Rtx-5MS column (30 m × 0.25 mm i.d., 0.25 μm film thickness) (Restek Corporation, Bellefonte, PA, USA). The carrier gas was helium (99.999%) at a flow rate of 1 mL min^−1^. The oven temperature was initially held at 40 °C for 1 min, then increased to 270 °C at a rate of 10 °C min^−1^ and held for 5 min. The injector temperature was maintained at 250 °C, while the ion source temperature was set at 230 °C. Samples were injected with a split ratio of 50:1, and the injection volume was 0.2 μL. Mass spectra were obtained using an electron ionization (EI) system at 70 electron volt with a quadrupole analyzer. Data acquisition and processing were performed using Agilent MSD ChemStation software (Rev E.02.02.1413). The chemical composition of the compounds was determined using the GC–MS system’s built-in mass spectral library.

### 4.4. Fruit Micromorphology

To evaluate fruit micromorphological and morphometric variations, ten fully mature fruits (mericarps) per population were randomly selected and examined under a stereomicroscope (Olympus Corporation, Tokyo, Japan). Digital photographs of each specimen were captured and quantitative traits (e.g., mericarp length and width, wing width, and stylopodium dimensions) were precisely measured using ImageJ version 1.46r (National Institutes of Health, Bethesda, MA, USA). Additionally, qualitative characteristics (e.g., mericarp shape, stylopodium morphology and presence/absence of trichomes) were systematically recorded. Terminology for fruit shape description and taxonomic characterization was performed according to Flora Iranica [7,45].

### 4.5. DNA Extraction, PCR Amplification, and Sequencing

Total genomic DNA was extracted from dried leaf samples using the Plant Genomic DNA Extraction Kit (GenAll Biotechnology Co., Seoul, South Korea) according to the manufacturer’s instructions. DNA quality and concentration were evaluated prior to PCR amplification. Three molecular markers, including the nuclear ribosomal internal transcribed spacer (ITS) and the plastid intergenic spacers *psb*A-*trn*H and *rpl*32-*trn*L, were amplified by polymerase chain reaction (PCR). The ITS region was amplified using the universal primer pair ITS-U1 (5′-GGAAGKARAAGTCGTAACAAGG-3′) and ITS-U4 (5′-RGTTTCTTTTCCTCCGCTTA-3′) [46]. The plastid *psb*A-*trn*H spacer was amplified using the primers *psb*A-F (5′-GTTATGCATGAACGTAATGCTC-3′) [47] and *trn*H (5′-CGCGCATGGTGGATTCACAATCC-3′) [48]. The *rpl*32-*trn*L intergenic spacer was amplified using the primer pair *rpl*32-F (5′ CAGTTCCAAAAAAACGTACTTC-3′) and *trn*L (5′CTGCTTCCTAAGAGCAGCGT-3′) [49]. PCR amplification was performed in a 20 μL reaction volume containing genomic DNA, 2X PCR Master Mix, 10 pmol of each primer and nuclease-free water. Thermal cycling conditions consisted of an initial denaturation at 95 °C for 5 min, followed by 35 cycles of denaturation at 95 °C for 30 s, primer annealing at 50 °C for 45 s, extension at 72 °C for 45 s, and a final extension at 72 °C for 7 min. Amplified PCR products were separated by electrophoresis on 1% agarose gel and sequenced using an Applied Biosystems ABI 3500 Genetic Analyzer (Applied Biosystems, Foster City, CA, USA).

### 4.6. Phylogenetic Analyses

The ITS, *psb*A-*trn*H, and *rpl*32-*trn*L sequences were aligned separately using ClustalX version 2.0 [50] with default alignment parameters and were subsequently inspected and manually adjusted in MEGA version 7 [51] to improve alignment accuracy. Insertions and deletions (indels) were treated as gaps during sequence analysis. The aligned loci were then concatenated into a combined dataset for phylogenetic analysis. The best-fit nucleotide substitution model for the combined dataset was selected using the Model Selection function implemented in MEGA version 7 based on the Bayesian Information Criterion (BIC). The Tamura 3-parameter (T92) substitution model showed the lowest BIC value and was therefore selected for phylogenetic reconstruction. A Neighbor-Joining (NJ) tree was generated using the T92 model, and branch support was assessed by 1000 bootstrap replicates.

### 4.7. Data Analysis

Thirteen populations of *H. persicum* were investigated to identify the profile of chemical compounds using GC–MS analysis. A total of 90 compounds were identified, and their contents were reported as percentages. Twenty one chemical compounds (α-Linolenic acid, Linoleic acid, Palmitic acid, Stearic acid, Myristic acid, Roughanic acid, Palmitoleic acid, Behenic acid, Lignoceric acid Nonane, 3-methyl-, 9,12,15-Octadecatrien-1-ol, (Z,Z,Z)-, Decan, Dodecane, Tridecane Tetradecane, Hexadecane, Octadecane, Undecane, Fumaric acid, Anethole, Neophytadiene) that were present with more than 1% in at least one population that were common among all populations were selected for multivariate and clustering analysis, including principal component analysis and correlation analysis that were performed using the Past software (version 5.3) [52]. The Pearson correlation coefficients were calculated to determine the relationships between the studied traits. In order to determine the highly variable characters among the populations, factor analysis using the principal component analysis (PCA) method was performed. Canonical Correspondence Analysis (CCA) was performed to assess the relationships between phytochemical composition (21 compounds) and environmental factors (Elev, MAT, MAP) using the cca() function from the vegan package version 2.6-2 [53] in R version 4.4.2 [54] (Supplementary Code S1). CCA is a constrained ordination method that combines correspondence analysis with multiple regression, allowing the direct analysis of relationships between chemical composition and environmental variables. Mean annual precipitation (MAP), mean annual temperature (MAT) were extracted from the WorldClim global climate database (version 2.1) at a resolution of 30 arc-seconds [43] using the Raster package [55] in R software version 4.4.2. Prior to multivariate analysis, all chemical and environmental variables were standardized using Z-score transformation (mean = 0, standard deviation = 1) to eliminate scale differences among variables measured in different units (percentage, meters, °C, and mm). The standardization was performed using the scale function in R. The statistical significance of the CCA model, individual terms, and axes was assessed using permutation tests with 999 permutations via the anova() function. Variance partitioning was performed to determine the proportion of total inertia explained by constrained (environmental) and unconstrained (residual) components. The eigenvalues and their contributions to the scaled Chi-square were extracted using cca_model$CCA$eig and cca_model$CA$eig (Supplementary Code S1). The CCA biplot was generated using PAST version 5.3 for visualization purposes. Heatmap cluster and correlation analysis for combined data of all populations were obtained using R software. Morphological characters were standardized before analysis to eliminate the effect of differences in measurement scales. Multivariate analyses, including Principal Component Analysis (PCA) and Cluster Analysis (CA), were performed using PAST version 5.3 [52]. PCA was used to evaluate the contribution of morphological characters to population differentiation, while hierarchical clustering based on Euclidean distance and Ward’s method was performed to visualize relationships among populations.

## 5. Conclusions

Overall, the integration of phytochemical, morphological, molecular, and ecological evidence revealed substantial intraspecific diversification among wild populations of *H. persicum*. The correspondence among these independent datasets, particularly the shared patterns observed in the Margoon–Komehr and Alashtar–Kamandan–Naran groups and the pronounced differentiation of Zeri, supports the existence of geographically structured population differentiation and suggests that some populations may represent distinct intraspecific lineages. The marked variation in chemical composition, together with differentiation in fruit morphology and molecular relationships, indicates that phytochemical and morphological characters can provide complementary evidence for recognizing population-level differentiation within *H. persicum*. Moreover, the associations between chemical composition and environmental gradients, particularly altitude, temperature, and precipitation, provide exploratory evidence that ecological heterogeneity may contribute to the differentiation of population-specific chemical profiles. Collectively, these findings demonstrate the value of integrating phytochemical, morphological, molecular, and ecological evidence for characterizing population diversification and evolutionary structure within *H. persicum*. Future studies should include repeated sampling across several growing seasons to assess the temporal stability of the observed phytochemical patterns. In addition, growing successive generations from fruits collected from the studied populations under common-garden conditions could help distinguish genetic differentiation from environmental plasticity.

## Figures and Tables

**Table 1:** GC-MS derived chemical compounds of 13 natural populations *H. persicum*. RT denotes for retention time.

No.	Compound	Class	Rt	Relative Percentage													
				Formula	Komehr	Margoon	Kamandan	Alashtar	Naran	Shemshak	Zeri	Silvana	Gilvan	Ovan	Tangehvashi	Dehbala	Fandoghlou
1	Propanedioic acid, dimethyl ester	Esters	6.484	C_5_H_8_O_4_	-	-	0.45	0.37	0.17	0.61	0.27	-	0.43	-	-	0.55	-
2	Nonane, 5-methyl-	Aliphatic hydrocarbons	7.216	C_10_H_22_	0.42	-	0.53	-	0.51	0.54	0.61	1.04	0.77	0.94	0.94	0.9	0.91
3	Nonane, 3-methyl-	Aliphatic hydrocarbons	7.508	C_10_H_22_	0.51	0.5	0.68	-	0.58	0.63	0.72	-	1	1.17	1.15	1.1	1.11
4	Levulinic acid, methyl ester	Esters	8.052	C_6_H_10_O_3_	-	0.58	-	-	-	-	-	-	-	-	-	-	-
5	Decane	Aliphatic hydrocarbons	8.292	C_10_H_22_	8.79	8.74	11.77	9.31	9.98	10.48	11.5	19.91	16.2	17.8	18.02	16.27	17.13
6	3-Heptene, 2,2,4,6,6-pentamethyl-	Aromatic hydrocarbons	8.475	C_12_H_24_	-	-	-	-	-	0.48	-	-	-	-	-	-	-
7	Fumaric acid	Dicarboxylic acids	8.99	C_4_H_4_O_4_	1.22	0.73	-	1.59	6.06	1.82	0.73	1.71	2.84	2.21	1.99	2.9	1.02
8	Limonene	Terpenes	9.15	C_10_H_16_	-	2.71	0.39	0.91	0.22	-	-	-	-	-	-	-	-
9	1,8-Cineole	Terpenes	9.242	C_10_H_18_O	-	-	-	-	-	-	-	-	-	-	-	-	0.77
10	Butanedioic acid, dimethyl ester	Esters	9.293	C_6_H_10_O_4_	-	0.19	-	0.12	0.19	0.24	-	0.64	-	-	-	-	-
11	gamma-Terpinene	Terpenes	10.003	C_10_H_16_	-	0.17	-	-	-	-	-	-	-	-	-	-	-
12	1-Octanol	Alcohols	10.34	C_8_H_18_O	-	-	-	0.13	-	-	-	-	-	-	-	-	-
13	Undecane	Aliphatic hydrocarbons	11.084	C_11_ H_24_	-	0.36	0.75	0.4	0.38	0.41	0.47	0.86	0.7	0.74	0.78	0.65	0.76
14	Linalool	Terpenes	11.176	C_10_H_18_O	-	0.18	-	-	-	-	-	-	-	-	-	-	-
15	Butanedioic acid, hydroxy-, dimethyl ester	Esters	11.994	C_6_H_10_O_5_	-	0.18	-	-	-	-	0.75	-	-	-	-	1.98	0.5
16	Thiophene, 2-methyl-5-propyl-	Aromatic hydrocarbons	12.761	C_8_H_12_S	-	-	-	-	-	-	-	-	-	-	-	-	0.78
17	Butanedioic acid, methoxy-, dimethyl ester	Esters	12.595	C_7_H_12_O_5_	-	-	-	0.25	-	-	-	-	-	-	-	-	-
18	Undecane, 5-methyl-	Aliphatic hydrocarbons	12.664	C_12_H_26_	-	0.27	-	0.19	0.19	0.23	-	-	-	-	-	-	-
19	Undecane, 3-methyl	Aliphatic hydrocarbons	13.081	C_12_H_26_	0.2	-	-	0.55	-	-	-	-	-	-	-	0.37	-
20	Dotriacontan-1-ol	Alcohols	13.694	C_32_H_66_O	0.38	-	-	-	-	-	-	-	-	-	-	-	-
21	Dodecane	Aliphatic hydrocarbons	13.911	C_12_H_26_	4.21	4.28	5.86	4.83	4.86	5.37	6.13	11.16	8.85	9.12	9.27	8.71	9.32
22	Estragole	Phenylpropanoids	13.997	C_10_H_12_O	-	-	-	-	-	-	-	-	-	0.73	-	-	-
23	2-(4-Methoxyphenyl) ethanol	Alcohols	15.255	C_9_H_12_O_2_	-	-	-	2.34	-	0.21	-	-	-	-	-	-	-
24	Trans-anethole	phenylpropanoids	15.519	C_10_H_12_O	-	-	-	-	1.36	-	1.16	4.79	3.7	-	-	-	-
25	Anethole	Phenylpropanoids	16.44	C_10_H_12_O	-	-	-	-	-	0.71	16.6	-	-	-	0.92	1.17	0.72
26	Tridecane	Aliphatic hydrocarbons	16.646	C_13_H_28_	-	0.51	0.65	0.53	0.55	0.71	0.59	1.29	0.94	1.02	1.04	0.98	1.08
27	2,4-Decadienal, (E,E)-	Aldehydes	16.869	C_10_H_16_O	0.79	-	-	-	-	-	0.37	-	-	-	-	-	-
28	1-(4-Methoxyphenyl)-1-methoxypropane	Aromatic ethers	17.642	C_10_H_14_O_2_	-	-	-	-	-	-	-	-	1.74	-	-	-	-
29	Triacetin	Esters	18.088	C_9_H_14_O_6_	-	-	-	-	0.96	-	-	-	-	-	-	-	-
30	2-Undecenal	Aldehydes	18.128	C_11_H_20_O	-	-	-	-	-	-	0.48	-	-	-	-	-	-
31	Naphthalene, 1,2-dihydro-3,5,8-trimethyl	Aromatic hydrocarbons	18.237	C_13_H_16_	0.11	0.11	-	0.33	-	-	-	-	-	-	-	-	-
32	Naphthalene, 1,2,3,4-tetrahydro-1,6,8-trimethyl-	Aromatic hydrocarbons	19.072	C_13_H_18_	-	-	-	-	0.48	-	-	-	-	-	-	1.17	-
33	Benzene, 2-(2-butenyl)-1,3,5-trimethyl-	Aromatic hydrocarbons	19.084	C_13_H_18_	0.54	-	-	-	-	-	-	-	-	-	-	-	-
34	Benzene, 1,2,3,4-tetramethyl-4-(1-methylethenyl)-	Aromatic hydrocarbons	19.164	C_13_H_20_	-	0.2	-	-	-	0.53	-	-	-	-	-	-	-
35	Tetradecane	Aliphatic hydrocarbons	19.255	C_14_H_30_	1.94	2.1	2.62	2.28	2.44	2.68	2.87	5.64	4.4	4.23	4.61	4.27	4.64
36	Longicyclene	Terpenes	20.943	C_15_H_24_	-	-	-	-	-	-	0.29	-	-	-	-	-	-
37	Nonanedioic acid, dimethyl ester	Fatty acids/Esters	21.779	C_11_H_20_O_4_	-	-	-	-	-	-	-	-	0.44	0.84	0.55	-	0.76
38	Phenol, 2,6-bis(1,1-dimethylethyl)-	Phenolics	22.236	C_14_H_22_O	0.35	0.28	-	0.27	0.31	0.44	1.34	0.74	0.42	-	-	0.43	0.72
39	Lauric acid	Fatty acids	22.368	C_12_H_24_O_2_	-	-	-	-	-	0.19	-	-		-	-	0.51	-
40	Croweacin	Furan derivatives	22.471	C_11_H_12_O_3_	-	-	-	0.51	-	-	-	-	-	-	-	-	-
41	Benzene, 1,2-dimethoxy-4-(2-propenyl)-	Aromatic hydrocarbons	22.728	C_11_H_14_O_2_	-	-	-	2.33	-	-	-	-	-	-	-	-	-
42	Cis-Alpha-Bisabolene	Terpenes	22.871	C_15_H_24_	-	-	-	-	-	-	-	-	-	-	-	-	1.01
43	Nonanedioic acid, dimethyl ester	Esters	22.974	C_11_H_20_O_4_	-	-	-	-	-	-	-	-	0.44	0.84	0.55	-	0.76
44	Azelaic acid dimethyl ester	Fatty acid/Esters	22.974	C_11_H_20_O_4_	-	-	-	-	-	-	-	1.14	-	-	-	0.81	-
45	Hexadecane	Aliphatic hydrocarbons	24.062	C_16_H_34_	1.53	0.79	1.66	1.19	0.93	1.47	1.66	2.51	2.09	-	2.03	2.33	1.8
46	Pentadecane, 2,6,10-trimethyl-	Terpenes	25.16	C_18_H_38_	2.27	-	-	-	1.54	-	-	-	-	0.97	-	-	-
47	Heptacosane	Aliphatic hydrocarbons	25.475	C_27_H_56_	-	-	-	-	0.27	-	-	-	-	-	-	-	-
48	Dotriacontane	Aliphatic hydrocarbons	25.481	C_32_H_66_	0.38	1.06	-	-	-	-	-	-	-	-	-	-	-
49	1-Heptadecanol	Alcohol	25.876	C_17_H_36_O	-	0.4	-	-	-	-	-	-	-	-	-	-	-
50	Heptadecane	Aliphatic hydrocarbons	26.299	C_17_H_36_	4.9	-	-	0.34	-	-	0.58	-	0.47		-	0.55	1.38
51	Hexadecane, 2,6,11,15-tetramethyl-	Terpenes	26.408	C_20_H_42_	-	-	-	-	-	-	-	-	-	-	0.67	-	-
52	Pentadecane, 2,6,10,14-tetramethyl-	Aliphatic hydrocarbons	26.413	C_19_H_40_	3.57	-	-	-	2.1	-	-	-	-	1.58	-	-	-
53	Tetracosane, 2,6,10,15,19,23-hexamethyl-	Aliphatic hydrocarbons	26.425	C_30_H_62_	-	-	-	-	-	0.33	-	-	-	-	-	-	-
54	α-Pinene	Terpenes	26.614	C_10_H_16_	-	-	-	-	-	-	-	-	-	-	-	-	0.59
55	Myristic acid	Fatty acids	26.9	C_14_H_28_O_2_	-	0.61	1.53	0.56	-	0.97	1.11	2.35	1.39	0.87	1.87	2.54	2.22
56	Angelicin	Furanocoumarins	28.256	C_11_H_6_O_3_	-	-	-	-	-	0.51	-	-	-	-	-	-	-
57	Octadecane	Aliphatic hydrocarbons	28.428	C_18_H_38_	0.5	0.47	-	0.56	0.7	0.74	0.57	1.06	1.15	1.21	1.59	-	1.39
58	Tetradecanoic acid, 12-methyl-, methyl ester	Fatty acids/Esters	29.005	C_16_H_32_O_2_	0.24	-	-	-	0.78	-	-	-	-	-	-	-	-
59	Butanoic acid, 3,7-dimethyl-6-octenyl ester	Fatty acids/Esters	29.257	C_14_H_26_O_2_	0.81	-	-	-	-	-	-	-	-	-	-	-	-
60	Neophytadiene	Aliphatic hydrocarbons	29.761	C_20_H_38_	0.53	0.48	-	1.16	0.38	-	0.73	0.87	0.91	-	0.81	0.97	1.01
61	Benzene, 1-methoxy-4-(1-propenyl)-	Aromatic hydrocarbons	29.818	C_10_H_12_O	-	-	-	-	-	-	-	-	-	-	-	-	-
62	Phthalic acid, butyl cyclohexyl ester	Esters	29.984	C_18_H_24_O_4_	-	-	-	-	0.27	-	-	-	-	-	-	-	-
63	Dibutyl phthalate	Esters	29.984	C_16_H_22_O_4_	-	-	-	-	-	-	-	-	-	-	0.67	-	-
64	1-Eicosanol	Alcohols	30.121	C_20_H_42_O	-	-	-	-	-	-	-		-	-	-	-	1.85
65	1-Octadecanol	Alcohols	30.127	C_18_H_38_O	-	1.34		-	-	-	-	-	-	-	-	-	-
66	4,7-Methano-1H-indene, octahydro-	Polycyclic Hydrocarbons	30.384	C_10_H_16_	0.1	-	-	-	-	-	-	-	-	-	-	-	-
67	Nonadecane	Aliphatic hydrocarbons	30.447	C_19_H_40_	-	-	-	-	-	-	-	-	-	-	0.46	-	-
68	9,12,15-Octadecatrien-1-ol, (Z,Z,Z)-	Alcohols	30.51	C_18_H_32_O	-	-	-	9.04	4.69	-	-	0.92	-	1.33	0.74	-	0.82
69	Roughanic acid	Fatty acids	30.522	C_16_H_26_O_2_	-	8.66	-	-	-	9.3	-	-	2.01	-	-	-	-
70	Palmitoleic acid	Fatty acids	30.94	C_16_H_30_O_2_	-	1.82	-	-	1.7	1.84	2.3	1.4	1.92	2.2	-	1.86	-
71	Palmitic acid	Fatty acids	31.014	C_16_H_32_O_2_	18.71	16.71	19.33	17.35	20.01	17.22	13.8	22.53	16.8	22.5	23.55	21.38	20.08
72	Eicosane	Aliphatic hydrocarbons	32.376	C_20_H_42_	-	-	-	-	0.36	-	-	-	-	-	-	-	-
73	Hexatriacontane	Aliphatic hydrocarbons	32.381	C_36_H_74_	-	0.26	-	-	-	0.23	-	-	-	-	-	-	-
74	Margaric acid	Fatty acids	32.925	C_17_H_34_O_2_	-	-	-	0.31	-	-	-	-	-	-	-	-	-
75	Anteisomargaric acid	Fatty acids	32.931		0.49	-	-	-	0.22	-	-	-	-	-	-	-	-
76	Isobergapten	Furocoumarins	33.257	C_12_H_8_O_4_	-	0.15	-	-	0.13	-	0.28	-	0.42	-	-	-	-
77	gamma-Undecalactone	Lactones	33.669	C_11_H_20_O_2_	-	1.97	-	-	-	-	-	-	-	-	-	-	-
78	Bergapten	Furanocoumarins	33.829	C_12_H_8_O_4_	-	0.28	-	-	0.29	0.41	-	0.61	3.9	-	-	-	-
79	Benzene, 1,2,3,4-tetramethyl-5-(1-methylethyl)-z	Aromatic hydrocarbons	34.001	C_13_H_20_	-	-	-	-	0.62	-	-	-	-	-	-	-	-
80	Linoleic acid	Fatty acids	34.207	C_18_H_32_O_2_	11.18	13.49	11.17	10.32	12.04	10.04	7.1	6.57	6.15	7.35	7.83	7.03	8.44
81	α-Linolenic acid	Fatty acids	34.344	C_18_H_30_O_2_	28.88	24.23	36.52	24.55	15.49	22.4	20.5	8.74	9.43	9.3	7.14	5.52	6.28
82	Stearic acid	Fatty acids	34.762	C_18_H_36_O_2_	1.2	0.88	1.75	1.29	2.43	1.04	1.04	1.7	1.98	2.8	2.78	2.05	2.69
83	Pimpinellin	Furocoumarins	35.048	C_13_H_10_O_5_	-	0.7	-	-	0.35	0.55	-	-	-	-	-	-	0.63
84	Oroselone	Furocoumarins	35.386	C_14_H_10_O_3_	-	0.55	-	-	-	0.82	0.49	-	0.97	-	-	-	1.11
85	Tritetracontane	Aliphatic hydrocarbons	35.992	C_43_H_88_	-	-	-	-	0.25	-	-	-	-	-	-	-	-
86	Isopimpinellin	Furocoumarins	36.999	C_13_H_10_O_5_	-	0.38	-	-	-	1.15	-	-	-	-	-	-	-
87	Arachidic acid	Fatty acids	38.206	C_20_H_40_O_2_	-	-	-	0.25	0.37	0.47	-	-	-	1.2	-	0.37	0.9
88	Behenic acid	Fatty acids	41.382	C_22_H_44_O_2_	-	0.41		-	-	0.63	-	-	1.24	1.76	2	0.58	1.06
89	Lignoceric acid	Fatty acids	44.323	C_24_H_48_O_2_	0.97	0.84	-	-	-	1.24	0.75	-	1.86	3.17	2.8	1.36	1.58
90	Cerotic acid	Fatty acids		C_26_H_52_O_2_	-	-	-	-	-	-	-	-	-	1.48	-	1.13	-
	Fatty acids/esters				61.99	67.65	70.30	54.63	51.34	65.34	46.60	44.43	43.22	53.47	48.98	45.14	44.01
	Aliphatic hydrocarbons				29.75	19.82	24.52	21.34	24.48	23.82	26.43	44.34	37.48	37.81	40.7	37.10	40.53
	Aromatic hydrocarbons				0.65	0.31	-	2.66	1.10	1.01	-	-	-	-	-	1.17	0.78
	Polycyclic Hydrocarbons				0.1	-	-	-	-	-	-	-	-	-	-	-	-
	Alcohols				0.38	1.74	-	11.51	4.69	0.21	-	0.92	-	1.33	0.74	-	2.67
	Esters				-	2.74	0.45	0.74	1.59	0.61	1.02	0.64	0.87	0.84	1.22	2.53	1.26
	Terpenes				2.27	2.73	0.39	0.91	1.54	-	-	-	-	0. 97	0.67	-	2.37
	Furanocoumarins				-	2.06	-	-	0.77	3.44	0.77	0.61	5.29	-	-	-	1.74
	Aldehydes				0.79		-	-	-	-	0.85	-	3.7	-	-	-	-
	Phenyl propanoids				-	-	-	-	1.36	0.71	17.76	4.79	-	0.73	0.92	1.17	0.72
	Dicarboxylic acids				1.22	0.73	-	-	6.06	1.82	0.73	1.71	2.84	2.21	1.99	2.90	1.02
	Furan derivatives				-	-	-	0.51	-	-	-	-	-	-	-	-	-
	Phenolics				0.35	0.28	-	0.27	0.31	0.44	1.34	0.74	0.42	-	-	0.43	0.72
	Aromatic ethers				-	-	-	-	-	-	-	-	1.74	-	-	-	-
	Lactones				-	1.97	-	-	-	-	-	-	-	-	-	-	-
	Total				95.72	96.75	95.66	94.16	95.16	97.64	95.79	98.18	95.56	97.36	94.76	90.44	95.82

**Table 2:** Morphometric characters of the fruits (mericarps) of 13 populations of *H. persicum*. Abbreviations: FL: Fruit Length; FW: Fruit Width; FL/FW: Fruit Length to Width Ratio; DOVL: Dorsal Outer Vitta Length; DIVL: Dorsal Inner Vitta Length; DVID: Difference between Dorsal Outer and Inner Vitta Lengths; DOVBW: Dorsal Outer Vitta Basal Width; DIVBW: Dorsal Inner Vitta Basal Width; SW: Stylopodium Width; CVL: Commissural Vitta Length; CVBW: Commissural Vitta Basal Width; WW: Wing Width; SA: Stylopodium Angle.

Population Name	Mericarp Shape	Stylopodium Shape	FL	FW	FL/FW	DOVL	DIVL	DVID	DOVBW	DIVBW	SW	CVL	CVBW	WW	SA
Alashtar	obovate	Conical	10.91 ± 0.43	6.69 ± 0.19	1.62 ± 0.8	5.77 ± 0.52	6.18 ± 0.64	0.82 ± 0.55	0.59 ± 0.09	0.42 ± 0.06	1.91 ± 0.1	4.77 ± 0.3	0.56 ± 0.07	0.63 ± 0.09	80.33 ± 6.15
Kamandan	ovate	Conical	11.32 ± 1.86	7.92 ± 1.01	1.42 ± 0.05	6.81 ± 0.97	6.52 ± 0.59	0.3 ± 0.43	0.85 ± 0.17	0.67 ± 0.06	2.48 ± 0.36	5.4 ± 1.08	0.98 ± 0.19	0.92 ± 0.16	81.20 ± 3.45
Margoon	ovate	Conical	10.63 ± 0.84	7.33 ± 0.43	1.44 ± 0.05	6.62 ± 0.28	6.47 ± 0.37	0.16 ± 0.11	0.79 ± 0.05	0.94 ± 0.11	2.59 ± 0.17	5.39 ± 0.15	0.95 ± 0.09	0.94 ± 0.09	86.02 ± 0.05
Komehr	obovate	Conical	9.92 ± 0.62	7.85 ± 0.53	1.26 ± 0.04	5.73 ± 0.18	5.85 ± 0.41	0.24 ± 0.12	0.50 ± 0.04	0.38 ± 0.08	1.94 ± 0.22	4.99 ± 0.35	1.04 ± 0.09	0.87 ± 0.09	80.14 ± 5.22
Zeri	ovate	Narrowly conical	13.33 ± 0.45	8 ± 0.3	1.66 ± 0.04	6.53 ± 0.71	6.89 ± 1.42	1.5 ± 0.99	0.91 ± 0.05	0.79 ± 0.12	2.08 ± 0.55	5.18 ± 0.21	1.03 ± 0.06	0.99 ± 0.05	71.74 ± 7.79
Dehbala	obovate	Low conical	11.77 ± 0.65	8.43 ± 0.26	1.39 ± 0.09	5.21 ± 0.55	6.44 ± 0.38	1.22 ± 0.65	0.66 ± 0.17	0.50 ± 0.08	1.9 ± 0.13	3.8 ± 0.2	0.89 ± 0.1	0.95 ± 0.16	100.49 ± 6.19
Fandoghlou	obovate	Low conical	10.29 ± 0.63	8.69 ± 0.69	1.18 ± 0.1	5.44 ± 0.53	5.43 ± 0.63	0.45 ± 0.4	0.82 ± 0.09	0.7 ± 0.13	1.15 ± 0.23	4.02 ± 0.31	0.86 ± 0.07	0.91 ± 0.07	106.23 ± 6.7
Ovan	obovate	Low conical	10.98 ± 0.58	8.20 ± 0.57	1.33 ± 0.05	5.39 ± 0.26	5.94 ± 0.45	0.55 ± 0.33	0.75 ± 0.11	0.5 ± 0.01	1.87 ± 0.17	3.57 ± 0.45	1 ± 0.14	0.9 ± 0.06	104.53 ± 5.73
Tangehvashi	obovate	Low conical	10.08 ± 0.43	8.08 ± 0.49	1.24 ± 0.03	5.78 ± 0.32	6.04 ± 0.34	0.48 ± 0.33	0.85 ± 0.11	0.61 ± 0.04	2.09 ± 0.2	4.5 ± 0.31	0.92 ± 0.11	1.01 ± 0.16	114.54 ± 9.88
Shemshak	obovate	Conical	11.83 ± 0.43	7.21 ± 0.3	1.63 ± 0.06	5.24 ± 0.77	6.08 ± 0.51	0.84 ± 0.52	0.79 ± 0.21	0.73 ± 0.27	2.01 ± 0.4	3.33 ± 0.13	0.92 ± 0.1	0.68 ± 0.07	93.36 ± 4.15
Naran	obovate	Conical	9.87 ± 0.91	6.63 ± 0.66	1.49 ± 0.13	5.32 ± 1.42	6.61 ± 0.58	0.57 ± 0.35	0.72 ± 0.12	0.78 ± 0.14	1.99 ± 0.18	3.89 ± 0.28	0.89 ± 0.14	0.65 ± 0.07	94.09 ± 9.08
Gilvan	obovate	Low conical	11.86 ± 1.5	8.35 ± 0.83	1.46 ± 0.04	6.78 ± 0.67	6.62 ± 0.63	0.16 ± 0.1	0.72 ± 0.07	0.64 ± 0.15	2.4 ± 0.59	5.82 ± 0.72	1.07 ± 0.06	0.99 ± 0.09	104.72 ± 1.93
Silvana	obovate	Conical	11.47 ± 0.33	5.92 ± 0.3	1.94 ± 0.11	5.45 ± 0.16	6.79 ± 0.51	1.34 ± 0.49	0.53 ± 0.04	0.34 ± 0.06	1.71 ± 0.06	2.81 ± 0.018	0.71 ± 0.05	0.57 ± 0.08	83.55 ± 6.97

**Table 3:** Geographic origins, altitude, latitude, and longitude of studied *H. persicum* populations. The average annual temperature and rainfall is extracted from Worldclim database.

Pop. Name	Site	Abbreviation	Voucher Number	Latitude	Longitude	Altitude	Mean Annual Temp. [°C]	Rainfall [mm/Year]
Komehr	Fars	Kom	TUH—50184	30.4667	51.8421	2415	11.72	230
Margoon	Fars	Mar	TUH—50185	30.4907	51.8939	2310	12.38	218
Kamandan	Lorestan	Kam	TUH—50186	33.2979	49.4226	2110	11.11	294
Alashtar	Lorestan	Ala	TUH—50187	33.9461	48.3177	1855	12.27	375
Naran	Kordestan	Nar	TUH—50188	35.1669	47.1497	1788	10.91	483
Shemshak	Tehran	She	TUH—50189	36.0312	51.4847	2825	5.25	254
Zeri	West Azarbaijan	Zer	TUH—50190	38.4249	44.5826	2022	7.28	407
Silvana	West Azarbaijan	Sil	TUH—50191	37.4354	44.8349	1581	9.66	477
Gilvan	West Azarbaijan	Gil	TUH—50192	38.4094	44.3759	2144	6.87	438
Ovan	Ghazvin	Ova	TUH—50193	36.5142	50.4564	2018	9.66	324
Tangehvashi	Tehran	Tan	TUH—50194	35.9003	52.7272	2580	8.15	205
Dehbala	Yazd	Deh	TUH—50195	31.6003	54.1241	2490	11.5	132
Fandoghlou	Ardabil	Fan	TUH—50196	38.4157	48.5715	1546	8.70	434

## Data Availability

Data is contained within the article.

## Supplementary Materials

The following supporting information can be downloaded at: https://www.mdpi.com/article/10.3390/plants15203200/s1, Figure S1. Principal component analysis based on all chemical compounds; Figure S2. Association between 21 chemical traits across all studied populations of *H. persicum*; Figure S3. PCA biplot of fruit morphological traits for 13 wild populations of *H. persicum*; Table S1. Canonical coefficients, eigenvalues and estimated variance explained for the first three CCA axes. Only the first three axes are shown; Table S2. Correlation of major components of *H. persicum* and environmental factors. Asterisks indicate significance levels (* *p* < 0.05, ** *p* < 0.01); Supplementary Code S1: CCA for *H. persicum*.
