# Supplementary material for: Integrative Phytochemical, Morphological and Molecular Analyses Reveal Intraspecific Diversification in Wild Iranian Populations of Heracleum persicum Desf. ex Fischer
Source: Plants (Basel). 2026 Oct 19;15(20):3200. doi: 10.3390/plants15203200 (PMC13708793; doi:10.3390/plants15203200)
Supplement: Supplementary file 1 [file plants-15-03200-s001.zip › plants-4554179-supplementary.pdf]

Supplementary Material

Table S1. Canonical coefficients, eigenvalues and estimated variance explained for the first three CCA axes. Only the first three axes are shown. The first two axes accounted for 26.15% of the total variance, and the three axes together accounted for 29.08%.

	CCA Sets of Environmental variables		
**Traits**	1	2	3
**Phytochemicals**	0.31	0.08	-0.01
a-Linolenic acid	0.18	0.00	-0.03
Linoleic acid	-0.01	-0.03	-0.04
Palmitic acid	-0.12	-0.11	-0.01
Stearic acid	-0.45	-0.10	-0.18
Myristic acid	0.13	1.15	0.12
Roughanic acid	-0.04	0.15	0.24
Palmitoleic acid	-0.62	0.29	-0.13
Behenic acid	-0.46	0.29	-0.17
Lignoceric acid	-0.28	0.13	-0.11
Nonane, 3-methyl-	0.86	-0.74	0.32
9,12,15-Octadecatrien-1-ol	-0.18	-0.07	-0.01
Decan	-0.21	-0.09	-0.00
Dodecane	-0.28	-0.11	0.01
Tridecane	-0.21	-0.09	0.00
Tetradecane	-0.11	0.01	0.01
Hexadecane	-0.28	-0.00	-0.03
Octadecane	-0.26	-0.13	-0.00
Undecane	0.36	0.05	0.32
Fumaric acid	-0.87	0.08	0.57
Anethole	-0.06	-0.23	-0.03
Neophytadiene			
**Populations**			
Komehr	1.24	0.26	-1.80
Margoon	1.03	3.13	-0.44
Kamandan	1.10	0.11	-2.11
Alashtar	2.15	-2.11	1.39
Naran	1.39	-1.22	1.82
Shemshak	0.58	3.20	0.26
Zeri	-1.89	0.34	7.37
Silvana	-0.71	-1.22	-0.90
Gilvan	-0.74	0.27	-0.68
Ovan	-0.71	-0.58	-1.12
Tangehvashi	-1.12	-0.68	-1.49
Dehbala	-1.11	-0.69	-0.50
Fandoghlou	-1.14	-1.01	-1.90
**Environmental factors**			
Elevation (Elev)	0.05	0.96	-0.27
Mean annual temperature (MAT)	0.66	-0.46	-0.58
Mean annual precipitation (MAP)	-0.07	-0.63	0.76
Eigenvalues	0.074	0.039	0.012
Estimated variance (%)	17.13	9.02	2.93
Cumulative variance (%)	17.13	26.15	29.08

Table S2. Correlation of major components of *H. persicum* and environmental factors. Asterisks indicate significance levels (*$P < 0.05$, **$P < 0.01$).

	1	2	3	4	5	6	7	8	9	10	11	12	13	14	15	16	17	18	19	20	21	22	23	24
1	1																							
2	0.7**	1																						
3	-0.52	-0.25	1																					
4	-0.67*	-0.39	0.75**	1																				
5	-0.57*	-0.66*	0.43	0.36	1																			
6	0.25	0.42	-0.4	-0.53	-0.22	1																		
7	-0.29	-0.26	-0.25	-0.14	-0.05	0.35	1																	
8	-0.62*	-0.44	0.46	0.67*	0.31	0	0.04	1																
9	-0.55	-0.46	0.37	0.55*	0.2	0	0.14	0.95**	1															
10	-0.48	-0.37	0.26	0.63*	0.32	-0.12	0.14	0.76**	0.8**	1														
11	0.09	0.24	-0.02	0.03	-0.37	-0.25	-0.26	-0.25	-0.36	-0.5	1													
12	-0.81**	-0.81**	0.68*	0.69**	0.79**	-0.39	0.07	0.61*	0.52	0.43	-0.29	1												
13	-0.81**	-0.84**	0.62*	0.63*	0.81**	-0.38	0.09	0.55*	0.46	0.37	-0.27	0.99**	1											
14	-0.78**	-0.71**	0.56*	0.59*	0.82**	-0.17	0.19	0.54	0.37	0.33	-0.16	0.91**	0.92**	1										
15	-0.83**	-0.82**	0.62*	0.62*	0.81**	-0.34	0.1	0.54	0.43	0.34	-0.26	0.99**	1**	0.93**	1									
16	-0.27	-0.48	0.1	-0.03	0.7**	-0.22	-0.21	-0.11	-0.19	-0.04	-0.3	0.4	0.45	0.35	0.49	1								
17	-0.95**	-0.68*	0.58*	0.7**	0.56*	-0.22	0.09	0.76**	0.7**	0.56*	-0.13	0.8**	0.79**	0.72**	0.81**	0.27	1							
18	-0.57*	-0.64*	0.52	0.61*	0.81**	-0.29	0.04	0.49	0.31	0.36	-0.18	0.86**	0.85**	0.93**	0.84**	0.33	0.53	1						
19	-0.35	0	0.23	0.34	-0.28	-0.12	0.39	0.03	-0.01	0.04	0.33	0	-0.01	0.03	0.02	-0.13	0.23	-0.1	1					
20	0.06	-0.29	-0.55	-0.31	0.01	-0.13	0.35	-0.21	-0.07	0.06	-0.18	-0.12	-0.09	-0.11	-0.11	0.1	-0.14	-0.08	-0.21	1				
21	-0.46	-0.39	-0.02	0.06	0.39	-0.37	-0.23	-0.02	-0.09	-0.12	0.3	0.27	0.33	0.23	0.36	0.56*	0.43	0.13	-0.03	0.12	1			
22	0.2	0.14	-0.09	-0.28	-0.11	0.52	0.1	0.25	0.35	0.29	-0.4	-0.25	-0.29	-0.28	-0.29	0.03	-0.02	-0.32	-0.01	-0.04	-0.33	1		
23	0.31	0.52	0.18	-0.08	-0.24	-0.25	-0.31	-0.41	-0.4	-0.35	0.39	-0.32	-0.33	-0.37	-0.35	-0.25	-0.36	-0.29	0.03	-0.34	0.12	-0.23	1	
24	-0.17	-0.21	-0.15	0.16	-0.07	-0.31	0.11	-0.22	-0.33	-0.3	0.35	0.16	0.21	0.23	0.22	0.01	-0.01	0.22	0.22	0.16	0.19	-0.82**	-0.22	1

1, α-Linolenic acid; **2**, Linoleic acid; **3**, Palmitic acid; **4**, Stearic acid; **5**, Myristic acid; **6**, Roughanic acid; **7**, Palmitoleic acid; **8**, Behenic acid; **9**, Lignoceric acid; **10**, Nonane, 3-methyl-; **11**, 9,12,15-Octadecatrien-1-ol, (Z,Z,Z)-; **12**, Decan; **13**, Dodecane; **14**, Tridecane; **15**, Tetradecane; **16**, Hexadecane; **17**, Octadecane; **18**, Undecane; **19**, Fumaric acid; **20**, Anethole; **21**, Neophytadiene; **22**, Elevation; **23**, Mean annual temperature (MAT); **24**, Mean annual precipitation (MAP).

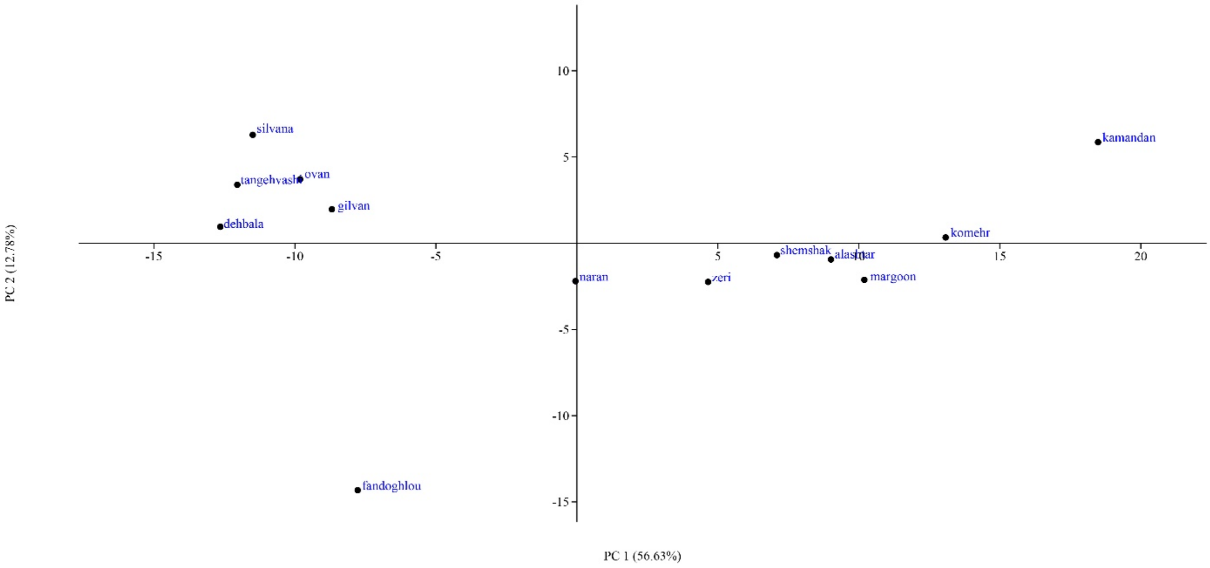

Figure S1. Principal component analysis revealing differences among populations based on all chemical compounds.

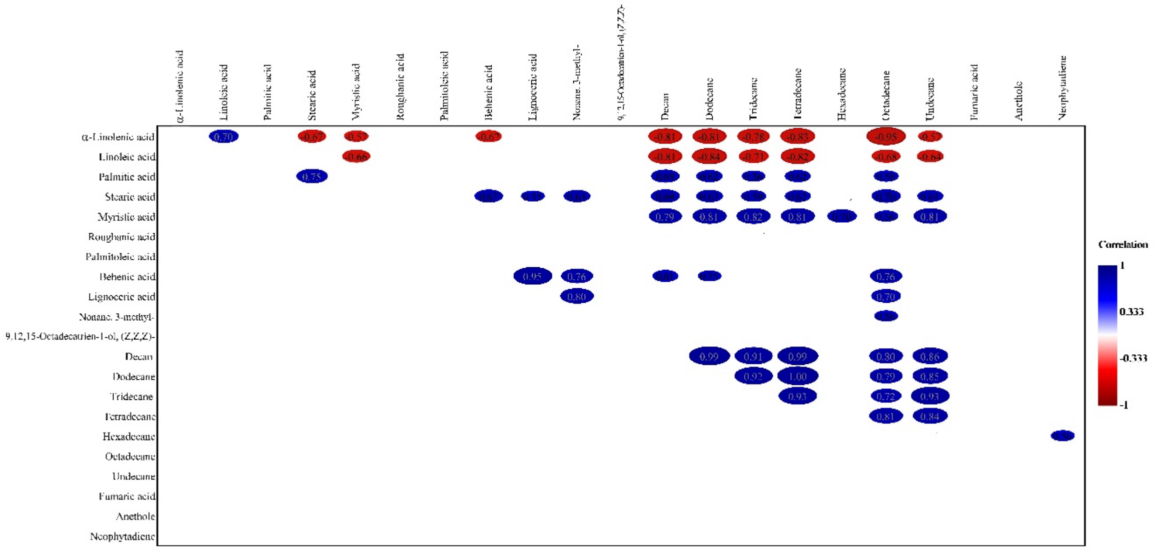

Figure S2. Association between 21 chemical traits across all studied populations of *H. persicum.*

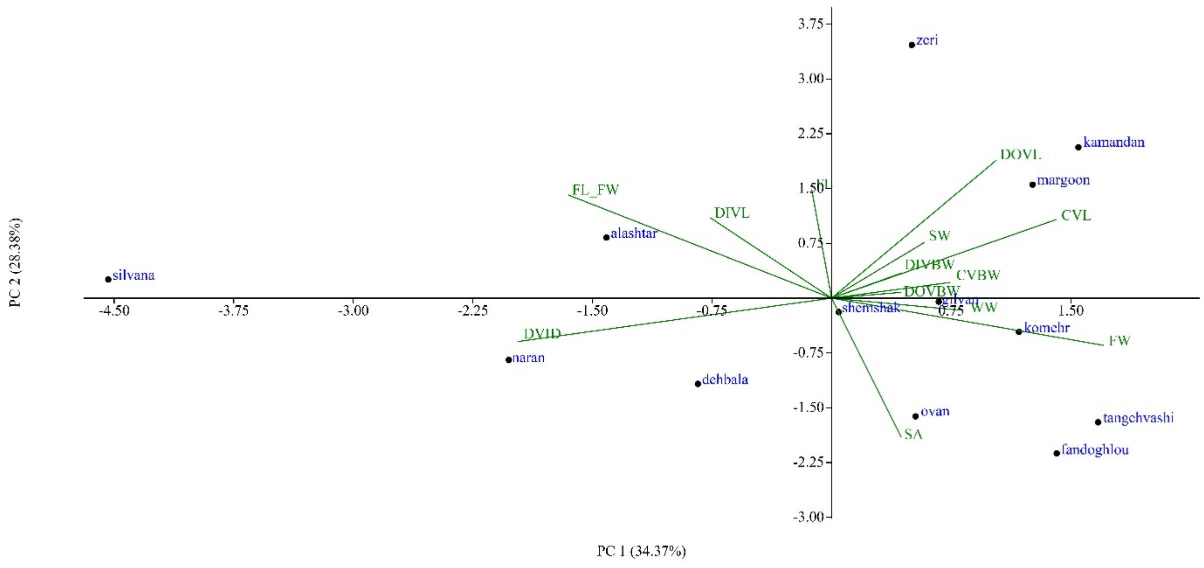

Figure S3. PCA biplot of seed morphological traits for 13 wild populations of *H. persicum*.

Software and Packages

All CCA analyses were performed in R version 4.6 (R Core Team, 2024). The following packages were used:

Package	Version	Purpose
vegan	2.6-4	CCA, permutation tests, variance partitioning
readxl	1.4.3	Reading Excel data
openxlsx	4.2.9	Writing Excel output

```
# Supplementary Code S1: CCA Analysis for Heracleum persicum

# --- 1. Load packages ---
library(readxl)
library(vegan)
library(openxlsx)

# --- 2. Read data ---
raw_data <- read_excel("CCA data.xlsx", sheet = "Sheet1", range = "A1:Y14")
chem_data <- as.data.frame(raw_data[, 2:22])
env_data  <- as.data.frame(raw_data[, 23:25])
rownames(chem_data) <- raw_data$Comound
rownames(env_data)  <- raw_data$Comound
chem_data <- as.data.frame(lapply(chem_data, as.numeric))
env_data  <- as.data.frame(lapply(env_data, as.numeric))
rownames(chem_data) <- raw_data$Comound
rownames(env_data)  <- raw_data$Comound

# --- 3. CCA with 21 compounds ---
cca_all <- cca(chem_data ~ Elev + MAT + MAP, data = env_data)
summary(cca_all)
```

```
# --- 4. Testing CCA with permutation ANOVA (999 permutations) ---
anova(cca_all, permutations = 999)
anova(cca_all, by = "term", permutations = 999)
anova(cca_all, by = "axis", permutations = 999)

# --- 5. Variance explained by CCA axes ---
eig_c <- cca_all$CCA$eig
var_c <- eig_c / sum(c(cca_all$CCA$eig, cca_all$CA$eig)) * 100
print(round(var_c[1:3], 2))

# --- 6. Extract scores for sites, environmental factors, and species ---
site_scores <- as.data.frame(scores(cca_all, display = "sites", scaling = 2, choices = 1:2))
site_scores$Site <- rownames(site_scores)

env_scores <- as.data.frame(scores(cca_all, display = "bp", scaling = 2, choices = 1:2))
env_scores$Variable <- rownames(env_scores)

chem_scores <- as.data.frame(scores(cca_all, display = "species", scaling = 2, choices = 1:2))
chem_scores$Compound <- rownames(chem_scores)

# --- 7. Table S1: CCA coefficients for compounds (3 axes) ---
chem_coef <- as.data.frame(scores(cca_all, display = "species", scaling = 2, choices = 1:3))
colnames(chem_coef) <- c("CCA1", "CCA2", "CCA3")
chem_coef$Compound <- c(
  "α-Linolenic acid", "Linoleic acid", "Palmitic acid", "Stearic acid",
  "Myristic acid", "Roughanic acid", "Palmitoleic acid", "Behenic acid",
  "Lignoceric acid", "Nonane, 3-methyl-", "9,12,15-Octadecatrien-1-ol",
  "Decan", "Dodecane", "Tridecane", "Tetradecane", "Hexadecane",
  "Octadecane", "Undecane", "Fumaric acid", "Anethole", "Neophytadiene"
)
write.csv(chem_coef, "Table3_CCA_Compounds.csv", row.names = FALSE)
```

```r
# --- 8. Table S1 (continued): CCA coefficients for environmental factors ---
env_coef <- as.data.frame(scores(cca_all, display = "bp", scaling = 2, choices = 1:3))
colnames(env_coef) <- c("CCA1", "CCA2", "CCA3")
env_coef$Factor <- rownames(env_coef)
write.csv(env_coef, "Table3_CCA_Environmental.csv", row.names = FALSE)

# --- 9. Table S2: Correlation matrix (21 compounds + 3 environmental factors) ---
cor_matrix <- cor(chem_data, env_data, use = "pairwise.complete.obs")
write.csv(round(cor_matrix, 3), "Table4_Correlation.csv", row.names = TRUE)
```
